# Human macrophages utilize a wide range of pathogen recognition receptors to recognize Legionella pneumophila, including Toll-Like Receptor 4 engaging Legionella lipopolysaccharide and the Toll-like Receptor 3 nucleic-acid sensor

**DOI:** 10.1371/journal.ppat.1009781

**Published:** 2021-07-19

**Authors:** Lubov S. Grigoryeva, Nicholas P. Cianciotto

**Affiliations:** Department of Microbiology and Immunology, Northwestern University Medical School, Chicago, Illinois, United States of America; University of Pennsylvania Perelman School of Medicine, UNITED STATES

## Abstract

Cytokines made by macrophages play a critical role in determining the course of *Legionella pneumophila* infection. Prior murine-based modeling indicated that this cytokine response is initiated upon recognition of *L*. *pneumophila* by a subset of Toll-like receptors, namely TLR2, TLR5, and TLR9. Through the use of shRNA/siRNA knockdowns and subsequently CRISPR/Cas9 knockouts (KO), we determined that TRIF, an adaptor downstream of endosomal TLR3 and TLR4, is required for full cytokine secretion by human primary and cell-line macrophages. By characterizing a further set of TLR KO’s in human U937 cells, we discerned that, contrary to the viewpoint garnered from murine-based studies, TLR3 and TLR4 (along with TLR2 and TLR5) are in fact vital to the macrophage response in the early stages of *L*. *pneumophila* infection. This conclusion was bolstered by showing that i) chemical inhibitors of TLR3 and TLR4 dampen the cytokine output of primary human macrophages and ii) transfection of TLR3 and TLR4 into HEK cells conferred an ability to sense *L*. *pneumophila*. TLR3- and TLR4-dependent cytokines promoted migration of human HL-60 neutrophils across an epithelial layer, pointing to the biological importance for the newfound signaling pathway. The response of U937 cells to *L*. *pneumophila* LPS was dependent upon TLR4, a further contradiction to murine-based studies, which had concluded that TLR2 is the receptor for *Legionella* LPS. Given the role of TLR3 in sensing nucleic acid (i.e., dsRNA), we utilized newly-made KO U937 cells to document that DNA-sensing by cGAS-STING and DNA-PK are also needed for the response of human macrophages to *L*. *pneumophila*. Given the lack of attention given them in the bacterial field, C-type lectin receptors were similarly examined; but, they were not required. Overall, this study arguably represents the most extensive, single-characterization of *Legionella*-recognition receptors within human macrophages.

## Author summary

Although *Legionella pneumophila* causes an often-fatal form of pneumonia known as Legionnaires’ disease, many of the key attributes of the human immune response to this pathogen are relatively obscure. Using a range of genetic and biochemical approaches, we identified molecular receptors that serve critical roles in the ability of human macrophages to recognize and respond to *L*. *pneumophila* infection. Contrary to long-standing conclusions from past murine-based studies, we found that among these key host factors are the so-called Toll-like receptors TLR3 and TLR4, with the latter being responsible for sensing *Legionella* LPS/endotoxin. To our knowledge, this study is the most-exhaustive, single-analysis of *Legionella*-recognition receptors within human macrophages. Moreover, it underscores the need to supplement murine-modeling with human cell-based analysis, and its findings have implications for understanding the immune response to other bacterial pathogens and possibly the management of Legionnaires’ disease.

## Introduction

*Legionella pneumophila* is a Gram-negative bacterium that flourishes in natural and man-made water systems [1–3]. In native environments, *L*. *pneumophila* grows in amoebae [4]. However, if aerosolized by man-made devices, *L*. *pneumophila* can be inhaled and then replicate within both resident alveolar macrophages and monocyte-derived macrophages that migrate into the infected lung [5]. *L*. *pneumophila* infection is most often manifest as a severe form of pneumonia known as Legionnaires’ Disease (LD) [4,6–9]. The elderly and immunocompromised patients are at greater risk for developing LD, with death occurring in approx. one of every ten afflicted individuals [10]. Alarmingly, the rate of LD has more than tripled in recent years in the US and the first incidence of person-to-person transmission has now been recorded [11,12]. However, the mechanistic causes of human susceptibility to serious *L*. *pneumophila* infection is still largely unclear, although they likely include the manner in which the macrophage responds to the pathogen [13].

Upon entering macrophages through phagocytosis, *L*. *pneumophila* evades the lysosomal degradation pathway and orchestrates the formation of a unique vacuole, which is known as the *L*. *pneumophila*-containing vacuole (LCV) [14,15]. Within the LCV, *L*. *pneumophila* grows to large numbers before lysing the spent macrophage host [16,17]. While resident macrophages do not restrict *L*. *pneumophila* growth, they do sense and respond to infection, which entails the activation of an inflammatory cytokine response, among other things [5,9,13,18]. In general, macrophages sense bacteria through pathogen recognition receptors (PRRs) that are activated by pathogen associated molecular patterns (PAMPS) [19]. This activation leads to a signaling cascade that allows for the production and release of cytokines and chemokines. These soluble host factors in turn recruit and activate a range of additional immune cells, including neutrophils, dendritic cells, and T-cells [16]. Key among the PRRs are the Toll-like receptors (TLRs), of which there are ten in humans [20]. TLR1, -2, -5, -6, and -10 are on the cell surface, while TLR-3, -7, -8, -9 occur on endosomal vesicles [13,20–24]. TLR4 is found on both the cell surface and endosomal vesicles [24]. Surface TLRs often bind bacterial cell wall components including lipopolysaccharide (LPS) and flagellin [20], and endosomal TLRs commonly recognize nucleic acid derivatives from the invading pathogens [24]. Binding to PAMPs causes the TLR proteins to dimerize, engage various adaptor proteins, and thereby ultimately activate NFκB and other transcription factors, which promote the upregulation of many genes including those encoding pro-inflammatory cytokines [20,24].

Earlier *L*. *pneumophila* studies predominantly examined macrophage interactions using mouse models. Some found that, during infection of murine macrophages, TLR2, TLR5, and TLR9 are required for an optimal cytokine response [16,25]. Others, using receptor-knockout mice or macrophages from such mice, reported that TLR3 and TLR4 are not involved in murine-sensing of *L*. *pneumophila* [26–32]. Also, the murine PRR that engages *L*. *pneumophila* LPS was described to be TLR2, rather than TLR4, which is most often the PRR linked to LPS [26,31,33,34]. These various findings have regularly appeared in reviews in the field [5,13,14,18,21,25]. Recently, we observed that shRNA knockdown (KD) of TIR domain-containing adaptor inducing interferon beta (TRIF) results in a dampening of the cytokine response from U937 cells infected with *L*. *pneumophila* [22]. TRIF is best known as an adaptor downstream of endosomal TLR3 and TLR4 [23], and U937 cells are a human cell line that is differentiated to a macrophage-like state and hence are widely used in *L*. *pneumophila* research and elsewhere [35–40]. Our results suggested that the importance of the different TLRs in the response of human macrophages to *L*. *pneumophila* may deviate significantly from their relevance in the murine macrophage-dependent response to that agent, and therefore, we argued that more should be done to characterize the response of human cells to *L*. *pneumophila* [22]. Indeed, in the context of *L*. *pneumophila*, relatively few other studies have considered the role of TLRs in human cellular responses, or in epidemiology-based findings on disease susceptibility [34,41–45]. Moreover, only one of these past studies directly showed the requirement of a TLR (i.e., TLR2) for a human macrophage response, which entailed exposure to purified bacterial outer membrane vesicles [43]. In the present study, we document that TLR3 and TLR4, along with TLR2 and TLR5, are in fact major PRRs for *L*. *pneumophila* recognition by human macrophages. Additionally, we demonstrate that TLR4, not TLR2, is the human receptor for *L*. *pneumophila* LPS and that a range of nucleic-acid sensing PRRs in addition to TLR3 are important in human macrophages. Thus, multiple aspects of the innate immune response that had gone unrecognized in past murine-based studies are now highlighted as being important during *L*. *pneumophila* infection of human cells.

## Results

### shRNA and siRNA KD of TRIF decreases the early cytokine response of human macrophages infected with virulent *L*. *pneumophila*

**Fig 1:**
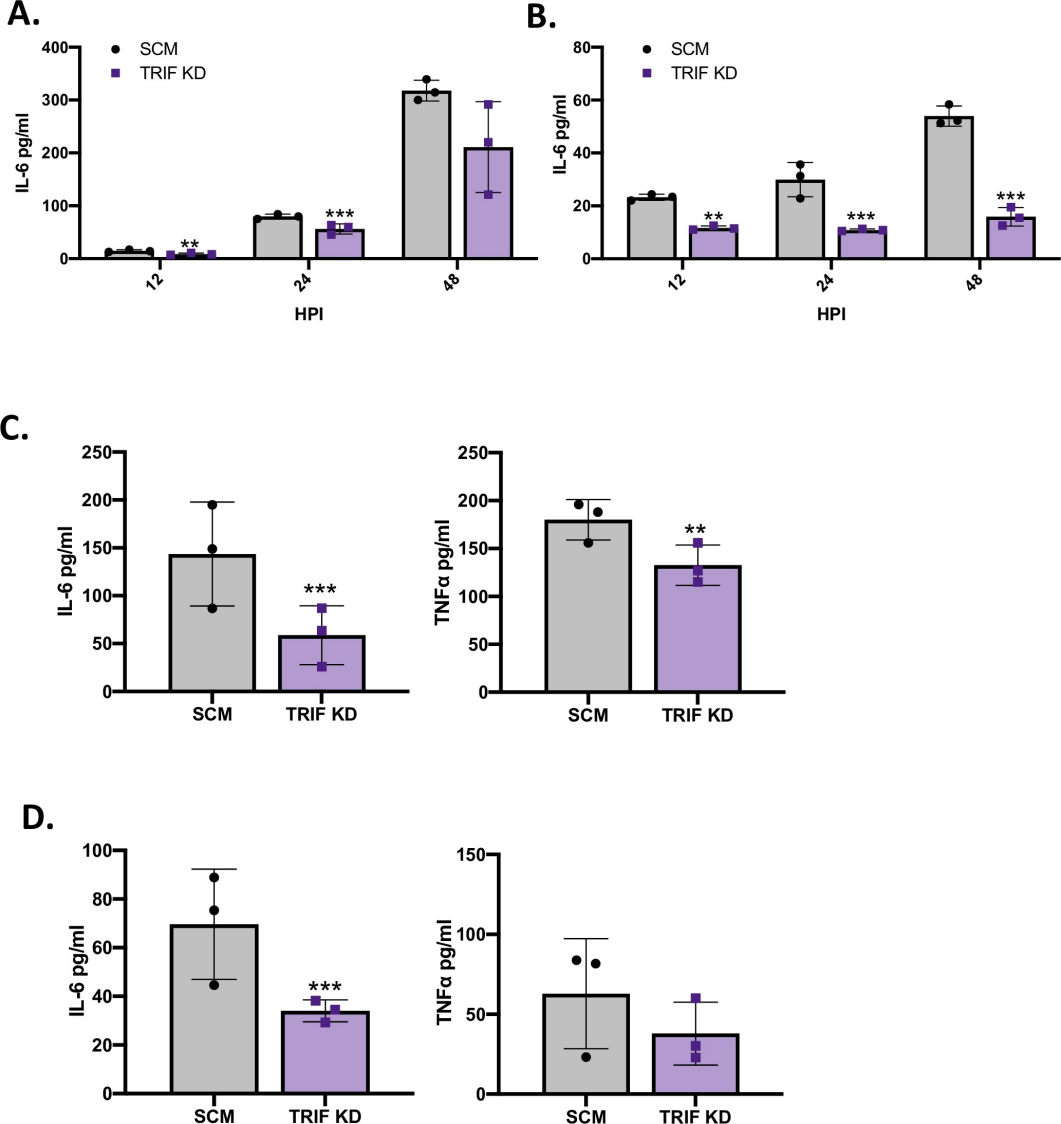
Effect of TRIF KD on cytokine production from human macrophages after infection with *L*. *pneumophila*. (A—B) U937 cell macrophages containing either an shRNA targeting TRIF (TRIF KD) or a non-targeting scramble shRNA (SCM) were infected with strain 130b (A) or Philadelphia-1 (B) at a multiplicity of infection (MOI) of 0.5, and the levels of IL-6 in culture supernatants at 12, 24, and 48 h post infection (HPI) were then determined by single-cytokine ELISA. (C) SCM and TRIF KD cells were infected with strain 130b at a MOI of 20, and the levels of IL-6 (left) and TNFα (right) in culture supernatants after 9 h of infection were ascertained by single-cytokine ELISA. (D) PBMC-derived macrophages containing either an siRNA targeting TRIF (TRIF KD) or a non-targeting scramble siRNA (SCM) were infected with strain 130b at a MOI of 20 for 9 h, and then the levels of secreted IL-6 (left) and TNFα (right) determined by ELISA. The graphs in (A–D) show the average cytokine levels pooled from three (A -C) or two (D) independent experiments, each done in technical triplicate, with standard errors. The cytokine levels (pg/ml) were calculated relative to serial dilution of recombinant cytokine controls. Asterisks indicate points at which the values for samples from KD cells were significantly different from those for samples from SCM cells (**P < 0.01, ***P < 0.001, by Student’s *t* test).

Although our previous study implicated TRIF, a downstream adaptor protein of endosomal TLRs, as being required for full IL-6 secretion following infection of human U937 cells by the clinical isolate *L*. *pneumophila* strain 130b [22], a more thorough examination was needed. We first confirmed that a second, newly-derived shRNA KD of TRIF (S1 Table) in U937 cell macrophages (S1A Fig) leads to a decrease in the production of IL-6 following *L*. *pneumophila* infection. This decrease was not only evident at 24 h post infection, as we had seen previously [22], but also after 12 h of infection (Fig 1A). We next documented that the TRIF-dependent IL-6 response was not an oddity of infection by strain 130b by infecting the TRIF KD cells with another clinical isolate of *L*. *pneumophila*, strain Philadelphia-1. Again, there was a significant reduction in IL-6 secretion after both 12 and 24 h of infection (Fig 1B). Both of the *L*. *pneumophila* strains grew equally well in the KD macrophages as compared to control macrophages (S2A and S2B Fig), indicating that the reductions in IL-6 seen in Fig 1A and 1B were not due to diminished bacterial numbers but rather altered recognition. In further trials, the TRIF-dependency of the IL-6 response was seen as early as 9 h after infection (Fig 1C, left panel), a data point that clearly reflects an “early” response of initially-infected macrophages and not secondary infections within the monolayer [46]. The importance of TRIF was also evident when simultaneously measuring TNFα (Fig 1C, right panel). Thus, this dataset affirmed that TRIF is indeed an important factor in the early response of U937 cells to intracellular infection by wild-type, virulent strains of *L*. *pneumophila*. By analyzing cytokine production using multiplex ELISA, we confirmed that IL-6 and TNFα are the two most-highly secreted cytokines (S1B Fig). Other cytokines produced, to a smaller, but still significant degree, were IL-1β, IL-8, and IL-10 (S1B Fig). Transcriptomic analysis showed that IL-6 is the most up-regulated cytokine (S1C Fig), compatible with past transcriptomic data from infections of primary human monocyte-derived macrophages [47]. Our transcriptomic analysis also confirmed TNFα and IL-1β as highly up-regulated cytokines, and further identified upregulated chemokines, including CSF3, CSF2, CXCL8, CLec4E, CD80 and CXCL10 (S1C Fig). Thus, in agreement with the types of cytokines that are found in the blood and tissue of humans with LD [9,13,48], we concluded that it is appropriate to continue to use IL-6 and TNFα as markers for the response to *L*. *pneumophila* infection. Importantly, siRNA KD of TRIF (S1 Table) in human peripheral blood mononuclear cells (PBMC)-derived macrophages (S3A Fig) led to an approx. 60% reduction in IL-6 production following *L*. *pneumophila* infection (Fig 1D, left panel), confirming the results that we had obtained utilizing U937 cells (Fig 1C). The impact of TRIF on TNFα production by the infected PBMC-derived macrophages was less apparent, trending toward statistical significance (Fig 1D, right panel). Together, these data support our hypothesis that TRIF has a critical role in the early stages of the human innate immune response to *L*. *pneumophila* infection.

### CRISPR/Cas9 KO of TRIF and MyD88 dampens the cytokine response of human macrophages to *L*. *pneumophila* infection

**Fig 2:**
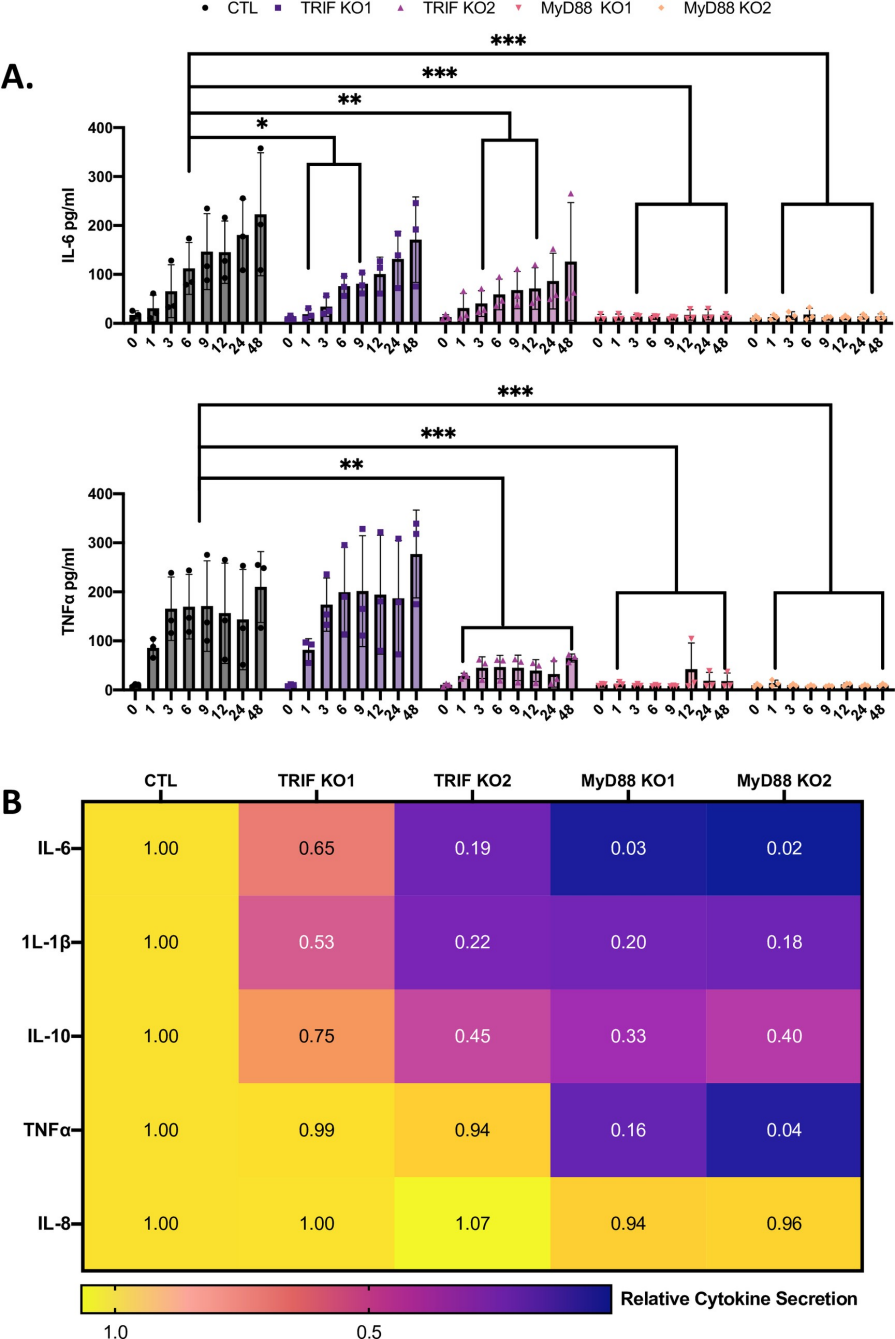
Effect of CRISPR/Cas9 KO of TRIF and MyD88 on cytokine production from human macrophages after infection with *L*. *pneumophila*. (A) U937 cell macrophages containing either a mutation in the TRIF gene (TRIF KO1, TRIF KO2) or MyD88 gene (MyD88 KO1, MyD88 KO2) or a non-targeting CRISPR Guide plasmid (CTL) were infected with strain 130b at MOI of 20, and levels of IL-6 (top) and TNFα (bottom) in culture supernatants obtained at 0, 1, 3, 6, 9, 12, 24, and 48 h (as indicated) were determined by ELISA. The cytokine levels (pg/ml) were calculated relative to serial dilution of recombinant cytokine controls, and the presented graphs show the average cytokine levels (n = 3) pooled from three independent experiments, with standard errors. Asterisks indicate points at which the values for samples from KO cells were significantly different from those for samples from control cells (*P < 0.05, **P < 0.01, ***P < 0.001, by Student’s *t* test). (B) CTL, TRIF KO, and MyD88 KO U937 cells were infected with *L*. *pneumophila* strain 130b at a MOI of 20, and the secreted levels of IL-6, IL-1β, IL-10, TNFα, and IL-8 at 9 h post infection were then ascertained by multiplex ELISA. Cytokine levels resulting from infection of the control were set to a value of 1, and the levels from the various infected KO cells were normalized to the value of the control. Multiplex represents analysis of three pooled biological replicates (*n = 3*) each done in technical triplicate.

In order to improve upon the observations that we had made by examining shRNA KD macrophages, we constructed CRISPR/Cas9 knockout (KO) of TRIF in U937 cells (S2 Table). To heighten the rigor of this inquiry, we analyzed two independent KO’s, i.e., TRIF KO1 and KO2. Moreover, we made, for the sake of comparison, two CRISPR/Cas9 KOs of myeloid differentiation primary response 88 (MyD88) (S2 Table), a downstream signaling molecule of cell-surface TLRs and some endosomal TLRs that is likely important for the recognition of *L*. *pneumophila*, based on murine studies [25,49–52]. None of these KO’s changed the extent of *L*. *pneumophila* growth in macrophages (S2C Fig). Yet, both KO’s of TRIF led to a significant reduction in IL-6 as measured at 9 h after infection (Fig 2A, top), in agreement with our shRNA-based experiments. Both also showed the effect at 3 h and 6 h post infection, early time points not examined before (Fig 2A, top). Similar to the observations we made when using PBMC-derived macrophages, the TRIF-dependency of the TNFα response was less consistent, as only KO2 had a significant reduction in TNFα secretion (Fig 2A, bottom). KO of MyD88 showed an even greater reduction in both IL-6 and TNFα secretion from 3 to 48 h post infection (Fig 2A). Thus, TRIF was predominantly required for a full cytokine response at early time points after intracellular infection, whereas MyD88 was required at both the early and later stages. Multiplex cytokine analysis confirmed that both TRIF and MyD88 are required for the full production of IL-6 whereas only MyD88 impacted TNFα secretion (Figs 2B and S4). Other cytokines identified as being affected by TRIF as well as MyD88 were IL-1β and IL-10 (Figs 2B and S4). In sum, through the use of CRISPR/Cas9 technology, we demonstrated that TRIF is a factor in the recognition of *L*. *pneumophila* by human macrophages, with its impact being particularly evident at the earlier stages of intracellular infection and through measurements of IL-6. Moreover, we showed that MyD88 is also required for a full cytokine response following *L*. *pneumophila* infection of human macrophages. Prior murine-based studies indicated that TRIF has a redundant role with MyD88 for both activation of NF-κB, as evidenced by downregulation of the interferon-γ receptor, and caspase-11 dependent responses [53,54].

### CRISPR/Cas9 KO of TLR3 and TLR4 as well as TLR2 and TLR5 reduces the cytokine output of human macrophages following infection by *L*. *pneumophila*

**Fig 3:**
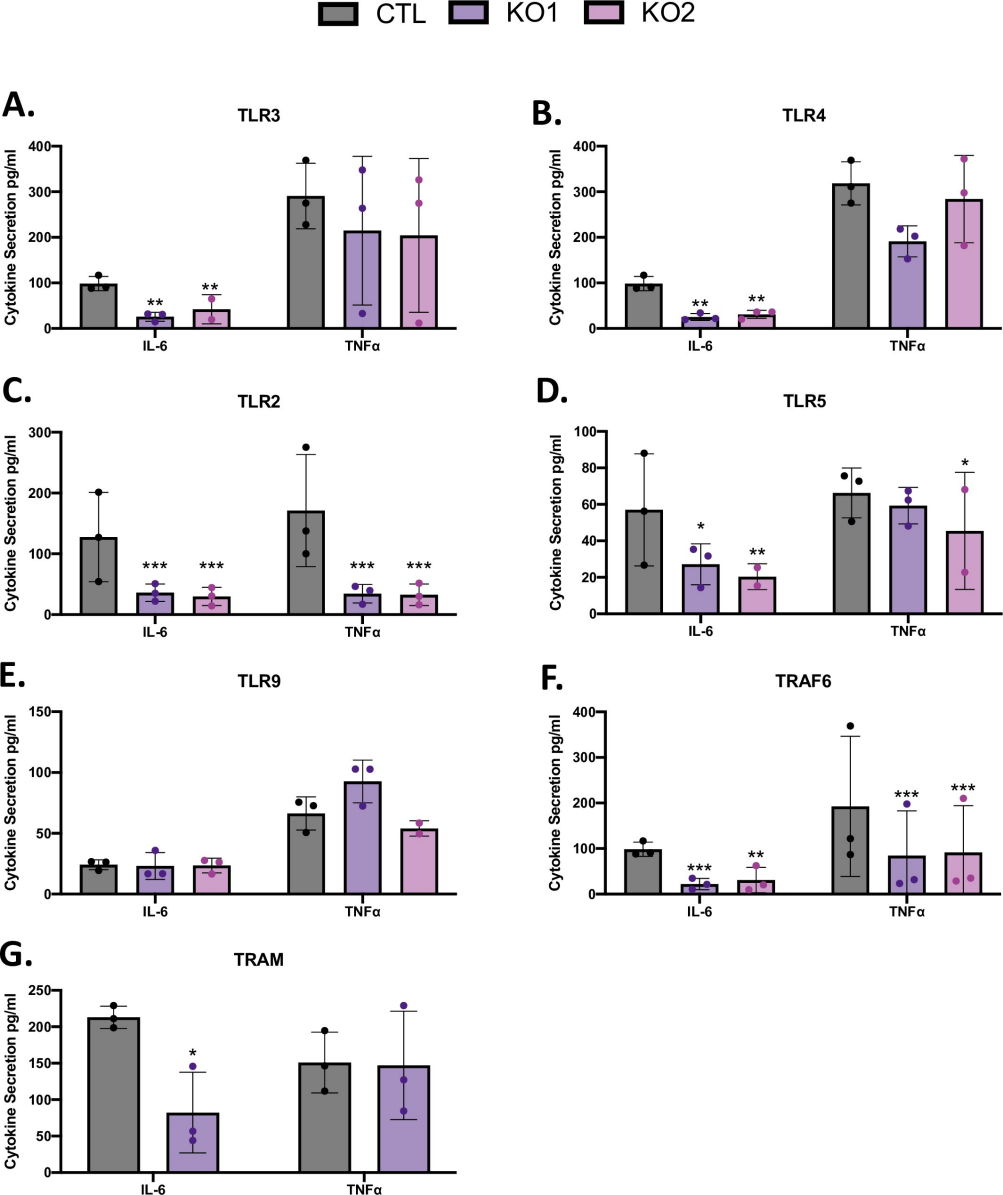
Effect of CRISPR/Cas9 KO of TLRs, TRAF6, and TRAM on cytokine production from human macrophages after infection with *L*. *pneumophila*. U937 cells expressing a non-targeting CRISPR guide plasmid (CTL, black bars) and independent clones of U937 cells containing a CRISPR/Cas9-generated mutation (KO1, KO2; purple and magenta bars) in either TLR3 (A), TLR4 (B), TLR2 (C), TLR5 (D), TLR9 (E), TRAF6 (F), or TRAM (G) were infected with strain 130b at a MOI of 20, and the levels of secreted IL-6 and TNFα at 9 h post infection were then determined by ELISA. The cytokine levels (pg/ml) were calculated relative to serial dilution of recombinant cytokine controls. Graphs show the average cytokine levels (*n* = 3) pooled from three independent experiments, done in technical triplicate, with standard errors. Asterisks indicate points at which the values for samples from KO cells were significantly different from those for samples from CTL cells (*P < 0.05, **P < 0.01, ***P < 0.001, by Student’s *t* test).

Given the novelty of our findings regarding TRIF, we sought to identify which TLR(s) upstream of TRIF is required for the early sensing of *L*. *pneumophila*. We pursued TLR3 and TLR4, the two TLRs that are associated with TRIF in other situations [23]. Whereas endosomal TLR3 is only linked to TRIF, TLR4, being endosomal and surface-localized, engages both TRIF and MyD88 [23]. Importantly, neither TLR3 nor TLR4 have heretofore been implicated as PRRs during experimental assessments of *L*. *pneumophila* infection. Quite to the contrary, prior studies using murine models concluded that TLR3 and TLR4 are not important in the immune response to *L*. *pneumophila* [26,27,29,49,55]. We utilized CRISPR/Cas9 technology to KO TLR3 and TLR4 in U937 cell macrophages, and as above, made two KO’s per target (S2 Table). As expected, KO of TLR3 resulted in decreased recognition of the known TLR3 agonist poly I:C (as measured by losses in IL-6 and TNFα secretion) but no significant impairments in response to the unrelated ligands/PAMPs *E*. *coli* LPS, PAM3CSK4 (PAM), flagellin, and CpG oligonucleotides (CpG) (S5A Fig). Also, as expected, KO of TLR4 caused less recognition of the known TLR4 agonist *E*. *coli* LPS but not poly I:C, PAM, flagellin, or CpG (S5B Fig). KO of TRIF only altered significantly the responses to poly I:C and LPS, as expected (S5C Fig). Compatible with the data obtained from analyzing the TRIF KD and KO (Figs 1 and 2), both TLR3 and TLR4 proved to be required for optimal production of IL-6 following infection with *L*. *pneumophila* (Fig 3A and 3B). In both cases, the KO gave an approx. 60–70% reduction in the cytokine’s output. Also, in agreement with the earlier experiments, TNFα seemed not to be impacted by the KO’s (Fig 3A and 3B). Given our data demonstrating the role of MyD88 in *L*. *pneumophila* infection of human macrophages, we constructed and characterized a panel of U937 cell CRISPR/Cas9 KO’s of TLR2, TLR5, and TLR9, the three MyD88-dependent TLRs that have been linked to *L*. *pneumophila* infection in murine models [26,49–52,56]. As expected, the TLR2 KO showed decreased recognition of the known TLR2 agonist PAM but was not significantly impaired in its response to the unrelated ligands poly I:C, *E*. *coli* LPS, flagellin, and CpG (S5D Fig), the TLR5 KO had less recognition of the known TLR5 agonist flagellin but not poly I:C, *E*. *coli* LPS, PAM, or CpG (S6A Fig), and the TLR9 KO was only consistently altered in its response to the known TLR9 agonist CpG (S6B Fig). Compatible with the behavior of the MyD88 KO (Fig 2), TLR2 and TLR5 proved important for both IL-6 and TNFα secretion following infection with *L*. *pneumophila* (Fig 3C and 3D). In contrast, KO of TLR9 did not change the production of either IL-6 or TNFα, indicating that, in human macrophages, TLR9 is not a relevant or important receptor during the early stages of intracellular infection (Fig 3E). Our prior shRNA KD of TLR9 in U937 cells also failed to reveal a required role for TLR9 at 24 to 72 h post infection [22]. Thus, these data indicated that TLR3 and TLR4, along with TLR2 and TLR5, play important roles in the human macrophage response to *L*. *pneumophila*. Moreover, the side-by-side comparison of the various KO cells (Fig 3A–3D) suggest that TLR3 and TLR4 are just as important for IL-6 secretion as TLR2 and TLR5. TNF Associated Factor 6 (TRAF6) is an adapter downstream of endosomal TLR3 and TLR4 as well as other immune signaling pathways, such as priming the NLRP3 inflammasome [57,58]. Therefore, we hypothesized that TRAF6 would also be required for cytokine production by *L*. *pneumophila*-infected macrophages. Indeed, CRISPR/Cas9 KO of TRAF6 in U937 cells dampened production of IL-6 and TNFα (Fig 3F). That the TRAF6 KO exhibited a greater overall reduction in cytokines compared to individual TLR KO’s affirms its role downstream of several immune-sensing pathways. Similarly, we found that KO of endosomal-TLR4 co-receptor, TRIF-related Adaptor Molecule (TRAM), led to a reduction in IL-6 (Fig 3G). Since ours were the first experiments to suggest a requirement for TLR3 and TLR4 in a macrophage response to *L*. *pneumophila* infection, we confirmed that the importance of these TLRs was evident over a range of MOI’s (S7 Fig). Moreover, we wanted to be sure that the findings in Fig 3A and 3B were not a peculiarity of infecting with strain 130b and thus assessed the relative ability of the TLR3 and TLR4 KO cells to recognize a range of *L*. *pneumophila* strains. U937 cells lacking either TLR3 or TLR4 showed impaired production of IL-6 upon infection with two other strains belonging to *L*. *pneumophila* serogroup-1 (S8A Fig) as well as during infection with three other strains representing *L*. *pneumophila* serogroups 7, 13, and 14 (S8B Fig). Furthermore, the importance of TLR3 and TLR4 was evident during intracellular infection by strains belonging to three other pathogenic species of *Legionella* (S8C Fig). Taken together, these data indicated that TLR3 and TLR4 are important PRRs during *L*. *pneumophila* infection of macrophages and in particular human macrophages.

### Transfected human TLR3 and TLR4 genes confer the ability to recognize *L*. *pneumophila*

**Fig 4:**
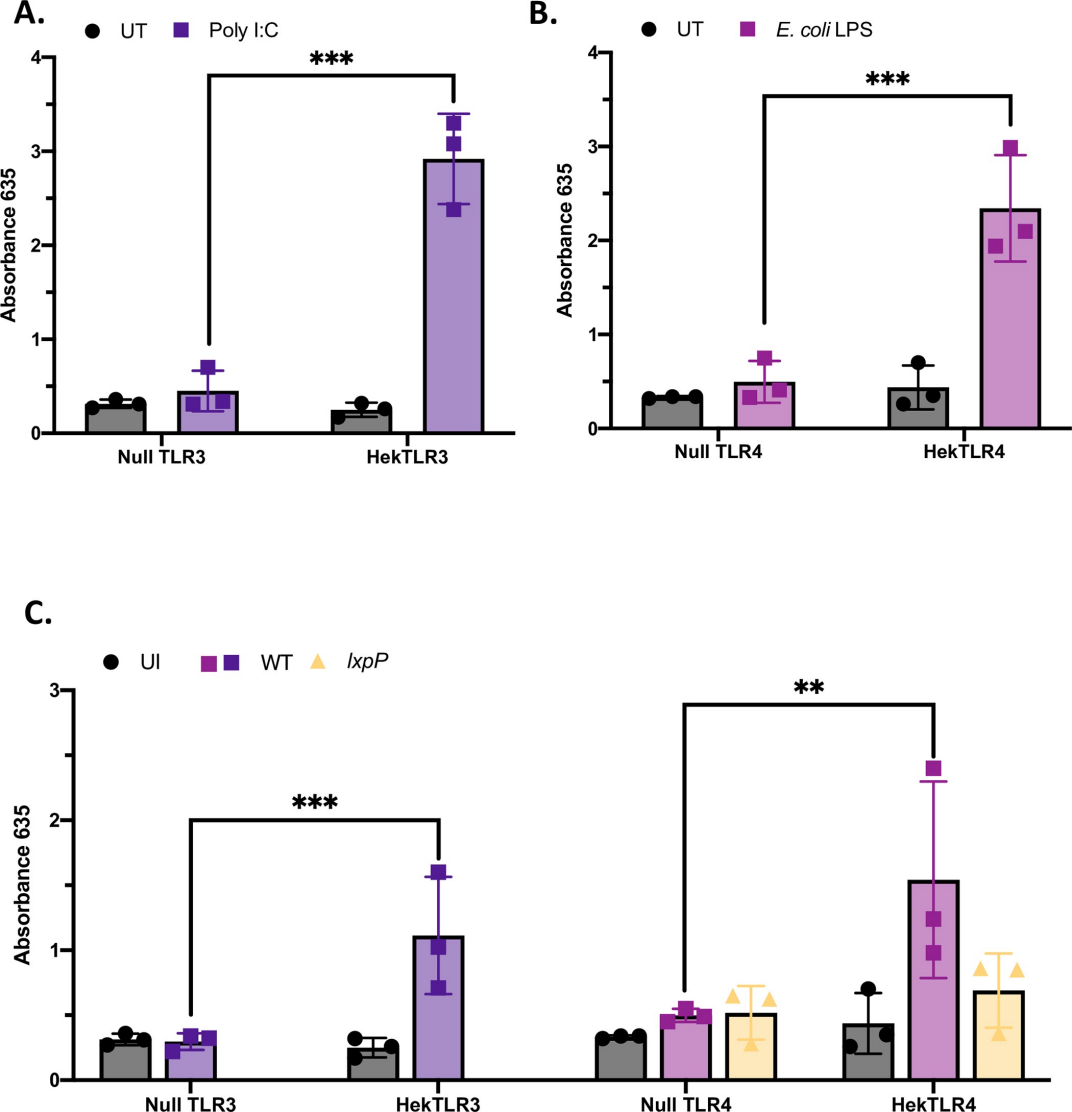
Effect of transfecting TLR3 and TLR4 on the ability of HEK293 cells to respond to *L*. *pneumophila* infection. (A-B) Hek-Blue cells expressing TLR3 (TLR3), Hek-Blue cells expressing TLR4 cells (TLR4), and their corresponding control cell lines not expressing the TLR (Null TLR3, Null TLR4) were treated with either vehicle control (UT), Poly I:C (A) or *E*. *coli* LPS (B) for 9 h and then the expression of an NF-kB-regulated promotor that is controlled by the levels of TLR activation was monitored via a fused alkaline phosphatase reporter gene. The enzyme produced by the reporter hydrolyzes a colorogenic substrate in the tissue culture medium, and therefore, the activation of the TLR is determined by changes in absorbance. (C) The various Hek-Blue cells were infected with *L*. *pneumophila* (Lpn) wildtype (WT) strain 130b or a *lpxP* mutant derivative (*lxpP*) at a MOI of 50 and assayed after 9 h for activation of TLR3 or TLR4, compared to uninfected control (UI), as described above. Results are presented as absolute absorbance read at 635. Values given are the average (*n = 3*) pooled from three independent experiments, done in technical triplicate, with standard errors. Asterisks indicate points at which the values for samples from TLR-transfected cells were significantly different from those for samples from null cells (*P < 0.05, **P < 0.01, ***P < 0.001, Student’s *t* test).

As an alternative means to assessing the ability of human TLR3 and TLR4 to recognize *L*. *pneumophila* infection, we sought to determine if a transfected TLR gene could confer upon a non-immune cell the capacity to recognize the bacterium. To begin, we confirmed that Hek-Blue cells that expressed TLR3 significantly responded to the known TLR3 agonist Poly I:C, as measured by the activation of a NF-κB-regulated promotor [59], when compared to a TLR-non-expressing (null) cell line [59,60] (Fig 4A). Similarly, we confirmed that Hek-Blue cells that expressed TLR4 and the activatable promotor [61] responded more significantly than null cells to the known TLR4 agonist *E*. *coli* LPS [61,62] (Fig 4B). Most importantly, when compared to their null controls, the Hek-Blue cells expressing either TLR3 or TLR4 responded to *L*. *pneumophila* infection (Fig 4C). Thus, our combined KO and transfection data indicated that TLR3 and TLR4 are both necessary and sufficient for the ability of human cells to sense *L*. *pneumophila*.

### Chemical inhibitors of TLR3 and TLR4 dampen the cytokine output of primary human macrophages but not murine macrophages in response to *L*. *pneumophila* infection

**Fig 5:**
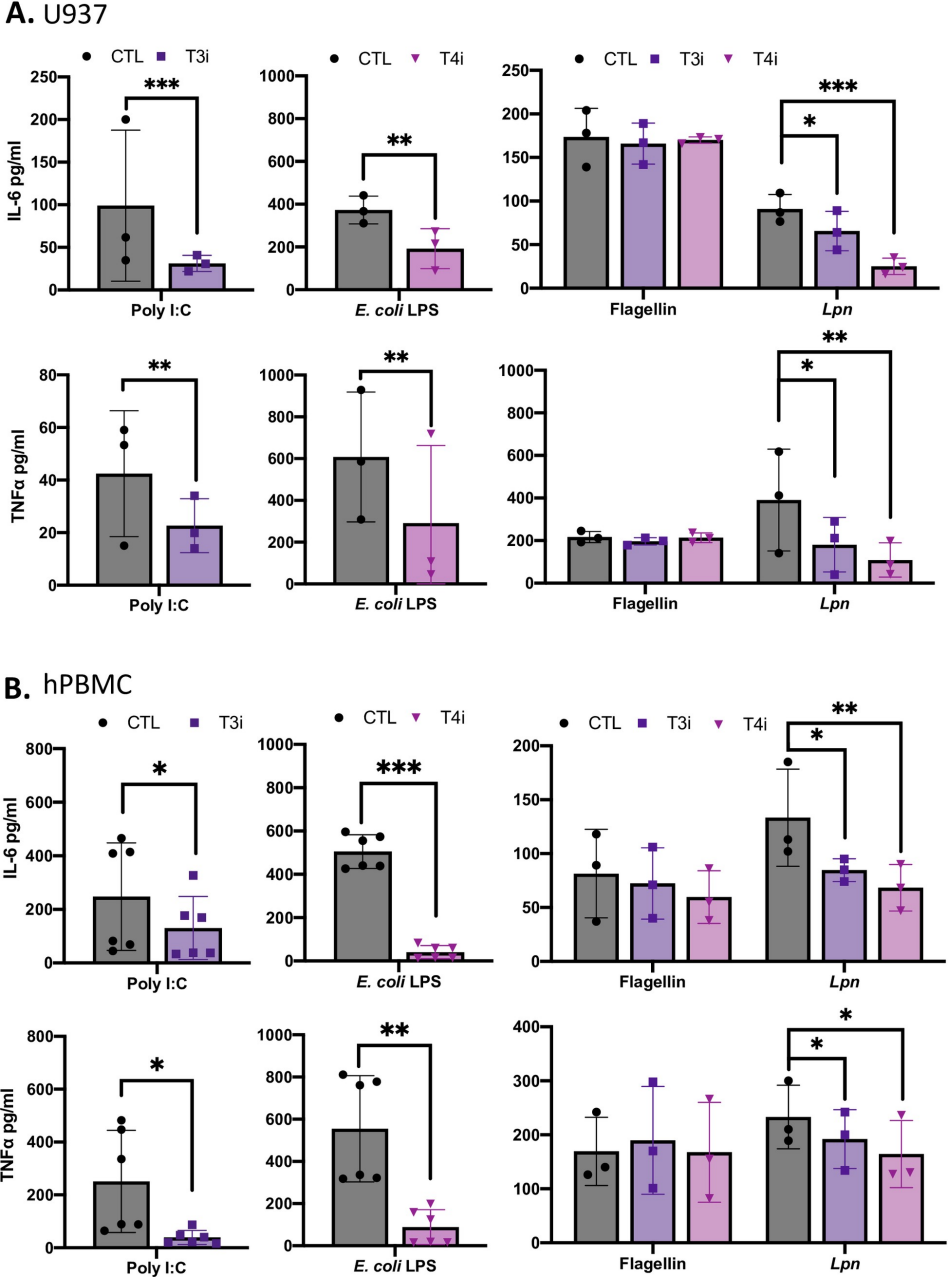
Effect of chemical inhibitors of TLR3 and TLR4 on the ability of human macrophages to produce cytokines following *L*. *pneumophila* infection. (A) U937 cells and (B) human PBMC-derived macrophages were pre-treated with either the TLR3 inhibitor Cu CPT 4a (T3i), TLR4 inhibitor TAK 242 (T4i), or vehicle control, DMSO (CTL). The macrophages were then exposed to either Poly I:C, *E*. *coli* LPS, or flagellin for 11 h, or infected for 9 h with strain 130b inoculated at a MOI 20 and levels of IL-6 and TNFα determined by ELISA. The cytokine levels (pg/ml) were calculated relative to serial dilution of recombinant cytokine controls. Graphs show the average cytokine levels (*n = 3*) pooled from three independent experiments, done in technical triplicate, with standard errors. Asterisks indicate points at which the values for samples from the inhibitor-treated cells were significantly different from those samples from CTL cells (*P < 0.05, **P < 0.01 ***P < 0.001, Student’s *t* test).

As a final means to gauge the importance of TLR3 and TLR4 and one that is not dependent on genetic manipulation, we asked whether chemical inhibitors of the TLRs would diminish the ability of the human macrophages to recognize *L*. *pneumophila*. To that end, we used the TLR3 inhibitor Cu CPT 4a [63] and the TLR4 inhibitor Tak-242 [64]. As expected, treatment of U937 cells with the TLR3 inhibitor led to a significant reduction in IL-6 and TNFα levels in response to Poly I:C [65] (Fig 5A, left panels). Similarly, U937 cell treatment with the TLR4 inhibitor led to a significant decrease in IL-6 and TNFα levels in response to *E*. *coli* LPS [66] (Fig 5A, center panels). Whereas neither of these inhibitors affected the host cell’s response to flagellin, a PAMP that does not signal through either TLR3 or TLR4, each inhibitor impaired the macrophage’s ability to recognize *L*. *pneumophila*, whether measured by levels of IL-6 or TNFα (Fig 5A, right panels). To validate these observations made with U937 cells, we tested the effect of the inhibitors on the ability of primary human PBMC-derived macrophage to recognize *L*. *pneumophila*. While there were still no off-target effects as evidenced by testing responses to flagellin, both inhibitors decreased IL-6 and TNFα levels in response to infection and control agonists (Fig 5B). Thus, chemical inhibition combined with genetic approaches affirm that TLR3 and TLR4 are critical for full recognition of *L*. *pneumophila* by human macrophages, including a standardly-used cell line and primary macrophages.

**Fig 6:**
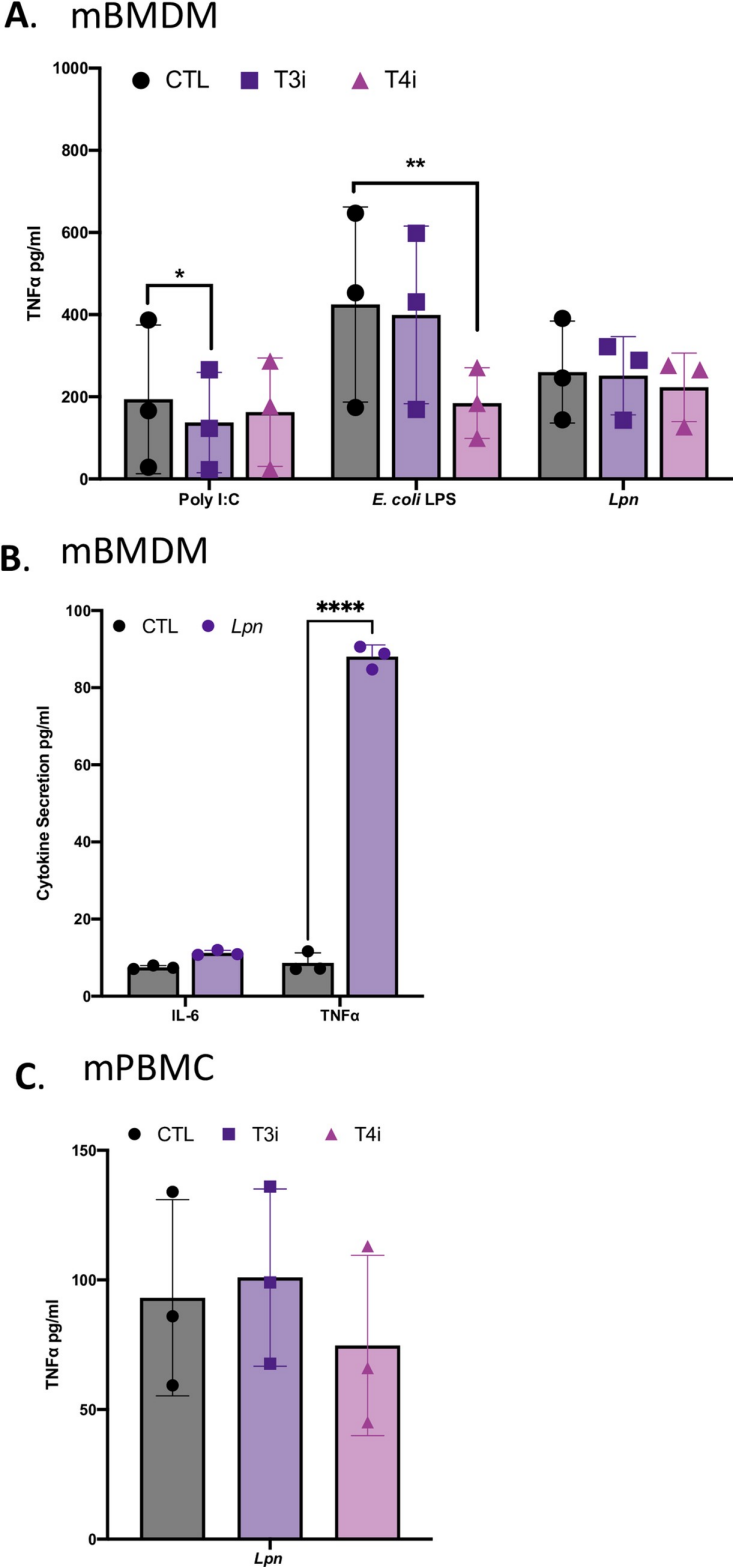
Effect of chemical inhibitors of TLR3 and TLR4 on the ability of murine macrophages to produce cytokines following *L*. *pneumophila* infection. **(**A) Murine BMDMs (mBMDM) were pre-treated with either the TLR3 inhibitor Cu CPT 4a (T3i), TLR4 inhibitor TAK 242 at (T4i), or the vehicle control, DMSO (CTL), and then, following either 11 h of exposure to Poly I:C or *E*. *coli* LPS or infection for 9 h with strain 130b inoculated at a MOI of 20, TNFα levels were determined by ELISA. The cytokine levels (pg/ml) were calculated relative to serial dilution of recombinant cytokine controls. (B) mBMDM were uninfected (CTL) or infected with strain 130b (*Lpn*), as indicated above, and their levels of secreted IL-6 and TNFα determined by ELISA and reported here as absolute values. (C) Murine PBMC-derived macrophages were pre-treated, infected with strain 130b, and then assayed for TNFα production, as described in (A). The graphs show the average cytokine levels (n = 3) pooled from three independent experiments, done in technical triplicate with standard errors. Asterisks indicate points at which the values for samples from inhibitor-treated cells (A, C) or *Lpn*-treated (B) were significantly different from those for samples from the CTLs (*P < 0.05, **P < 0.01, ****P < .0001, Student’s *t* test).

Interestingly, although the chemical inhibitors of TLR3 and TLR4 reduced the response of murine bone marrow derived macrophages (BMDMs) to the known agonists Poly I:C and *E*. *coli* LPS, they did not diminish the ability of those BMDMs to respond to *L*. *pneumophila* (Fig 6A). For these assays, we monitored levels of TNFα, since the murine macrophages did not significantly produce IL-6 after infection with *L*. *pneumophila* (Fig 6B). The TLR3 and TLR4 inhibitors also did not impede the ability of murine PBMCs to recognize *L*. *pneumophila* (Fig 6C). Thus, it appears that TLR3 and TLR4 are not critical in the innate immune response of murine macrophages to *L*. *pneumophila*. This may help explain why the importance of TLR3 and TLR4 that we have now identified had been missed in the many earlier *L*. *pneumophila* studies.

### TLR3- and TLR4-dependent cytokines promote the chemotaxis of neutrophil-like HL-60 cells

**Fig 7:**
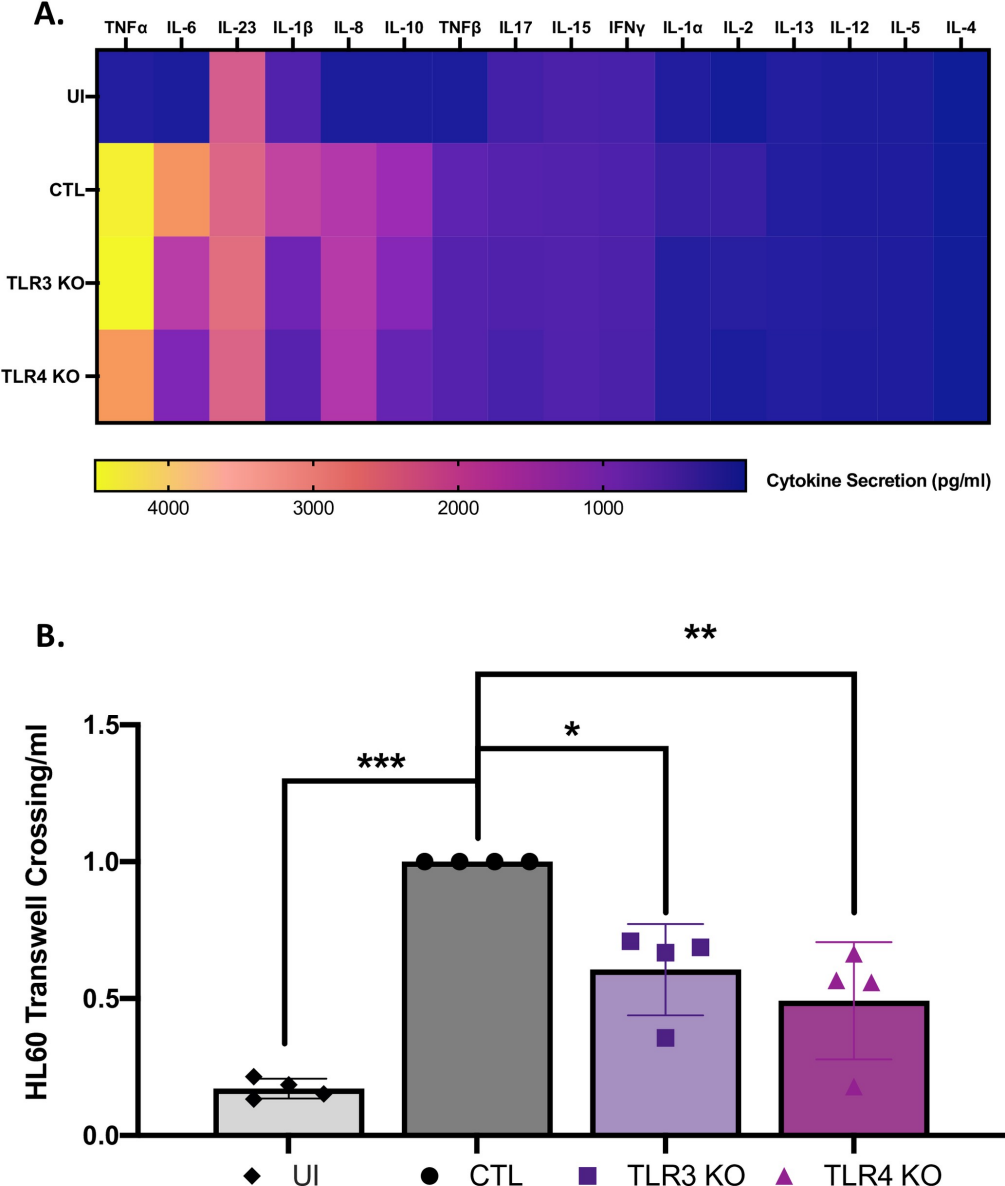
Effect of TLR3 and TLR4 on the production of various cytokines by human macrophages following *L*. *pneumophila* infection, and the chemotactic activity of that TLR3- and TLR4-dependent response. (A) U937 expressing a non-targeting CRISPR guide plasmid were either not infected (UI) or infected with strain 130b at a MOI of 20 (CTL), and the levels of sixteen different cytokines in culture supernatants were ascertained after 9 h by multiplex ELISA analysis, with the pg/ml levels presented using the indicated color scheme. U937 cells containing a KO of TLR3 or TLR4 (TLR3 KO, TLR4 KO) were similarly infected and assayed for cytokine production. Multiplex represents the analysis of three pooled biological replicates (*n = 3*) each done in technical triplicate. (B) Neutrophil-like HL-60 cells were exposed to the conditioned tissue culture media obtained from each one of the four macrophage populations noted above and then allowed to migrate across a monolayer of A549 cells into the lower chamber of a 3μm-transwell apparatus. The number of HL-60 cells crossing following exposure to the supernatants obtained from the CTL infection was set to a value of 1, and the levels of migration from the other treatments were normalized to the value of this control. The graph shows the average migration levels (*n =* 3) pooled from three independent experiments, done in technical triplicate, with standard errors. Asterisks indicate points at which the values were significantly different from those for samples from the CTL (*P < 0.05, **P < 0.01 ***P < 0.001, Student’s *t* test).

Upon doing multiplex ELISA that included sixteen cytokines, we confirmed that IL-6, along with IL-1β and IL-10, were most affected by the KO of TLR3 or TLR4 (Fig 7A). This was compatible with the results from multiplex ELISA analysis of the TRIF KO’s (Fig 2B). Among the *L*. *pneumophila*-induced cytokines impacted by TLR3 and/or TLR4, IL-6 and IL-1β are associated with increased neutrophil infiltration to sites of infection [67–70]. We posited that the cytokine output of the *L*. *pneumophila*-infected KO cells, as contained within their conditioned medium, would be less able to trigger neutrophil movement. Hence, as an initial way to assess the biological role of the TLR3- and TLR4-mediated response to *L*. *pneumophila*, we compared the ability of culture supernatants taken from WT vs. KO U937 cells to stimulate the movement of neutrophil-like human HL-60 cells across an epithelial cell monolayer consisting of human A549 cells [71,72]. Whereas the conditioned medium from normal U937 cells that had been infected with *L*. *pneumophila* triggered substantial cell migration (compared to supernatants from uninfected U937 cells), both the infected TLR3-KO and infected TLR4-KO cells were impaired in their ability to stimulate this chemotaxis (Fig 7B). Thus, the TLR3- and TLR4-dependent response of the human macrophage produces at least one outcome that has implications for the pathogenesis of *L*. *pneumophila* infection. This outcome is likely due to the action of TLR3/4-dependent IL-6, TNFα, and/or IL-1β as well as neutrophil chemokine CSF2 and CXCL10 and neutrophil stimulating factor CSF3 [67,73–75] that appear to be upregulated during *L*. *pneumophila* infection, e.g., as in S1C Fig.

### Multiple nucleic-acid PRRs are required for the full recognition of *L*. *pneumophila* by human macrophages

**Fig 8:**
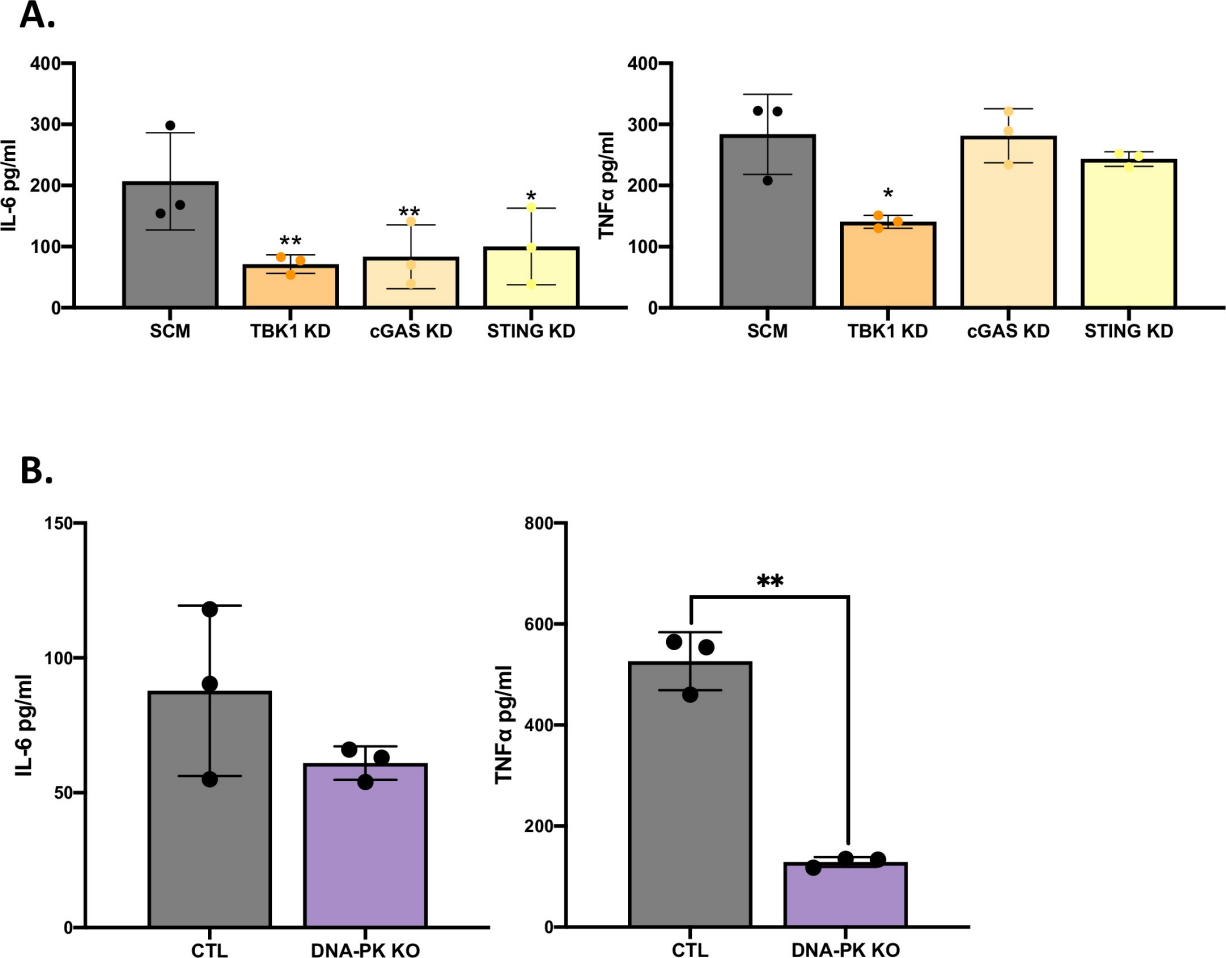
Effect of KD and KO of other nucleic acid-sensing pathways on cytokine production from human macrophages after infection with *L*. *pneumophila*. (A) U937 cell macrophages containing either an shRNA targeting TBK1 (TBK1 KD), cGAS (cGAS KD), or STING (STING KD) or a non-targeting scramble shRNA (SCM) were infected with strain 130b at a MOI of 20, and the levels of IL-6 (left panel) and TNFα (right panel) in culture supernatants at 9 h post infection were then determined by single-cytokine ELISA. The cytokine levels (pg/ml) were calculated relative to serial dilution of recombinant cytokine controls. (B) U937 cells expressing a non-targeting CRISPR guide plasmid (CTL, black bars) and U937 cells containing a CRISPR/Cas9-generated mutation in DNA-PK (purple bars) were infected with strain 130b for 9 h following a MOI of 20 and the levels of IL-6 (left) and TNFα (right) in culture supernatants were ascertained by ELISA. The cytokine levels (pg/ml) were calculated relative to serial dilution of recombinant cytokine controls. The graphs show the average cytokine levels (*n = 3*) pooled from three independent experiments, each done in technical triplicate, with standard errors. Asterisks indicate points at which the values for samples from KD or KO cells were significantly different from those for samples from SCM or CTL cells (* *P* < 0.05, **P < 0.01, by Student’s *t* test).

Using shRNA-mediated KD, we previously determined that TANK-binding kinase 1 (TBK1) has a major role in promoting IL-6 secretion by U937 cells infected with *L*. *pneumophila* for 24 to 72 h [22]. An Iκκ-related, serine/threonine kinase [76,77], TBK1 is an adaptor downstream of multiple PRRs, including the TLR3/TRIF, TLR4/TRIF/MyD88, RIG-I/MAVS, and cGAS/STING pathways [78–81]. Thus, we sought to determine if the cGAS/STING pathway is important for human macrophage recognition of *L*. *pneumophila*, potentially providing an additional explanation for the role of TBK1. The examination of cGAS/STING would examine the role of cytosolic dsDNA sensors [76, 82–85]. To more quickly begin this analysis, we utilized shRNA to KD in U937 cells (S3B Fig) both cGAS (DNA binder) and STING (adaptor), as well as using in parallel a previously confirmed KD of TBK1 [22] (S1 Table). As expected, the cGAS KD showed decreased recognition of the known agonist G3-ended Y-form Short DNA (G3-YSD) but was not significantly impaired in its response to the unrelated ligands poly I:C, *E*. *coli* LPS, PAM, and flagellin (S6C Fig). Moving on to *Legionella*, we kept our focus on the early stages of intracellular infection, in order to more easily make comparisons to the results obtained with the TRIF, MyD88, and TLR KD/KOs. In all three cases, the KDs diminished IL-6 production at 9 h after infection, and the magnitude of the effect was comparable to those tied to the TLRs (Fig 8A, left panel). In the case of the TBK1 KD, there was also an impact on TNFα (Fig 8A, right panel). These data indicated that human macrophages also utilize the cGAS-STING pathway to recognize *L*. *pneumophila*. Before we could do confirmatory experiments, including a second independent shRNA KD or a CRISPR/Cas9 KO of these factors, others reported on the importance of cGAS and STING for induction of cytokine transcription in human THP1 cells and human PBMC-derived macrophages following infection with *L*. *pneumophila* strains 130b and JR32 [86]. Thus, we felt it would be more fruitful to turn our attention to using CRISPR/Cas9 KO to test the role of another DNA-sensing pathway during *L*. *pneumophila* infection of human macrophages. DNA-PK is cytoplasmic, DNA-dependent protein kinase that is important during cellular DNA repair processes [87,88]. Although DNA-PK has been linked to viral and bacterial DNA-dependent cGAS/STING activation and STING-independent pathogen recognition pathways [88–90], it has never before been examined for a role in *Legionella* infection. Following the construction of a new U937 KO (S2 Table) and confirmation that this DNA-PK KO had impaired recognition of the known agonist CpG but not the unrelated poly I:C, *E*. *coli* LPS, PAM, and flagellin (S6D Fig), we observed that DNA-PK was important for TNFα, but not IL-6, production by *L*. *pneumophila*-infected macrophage at 9 h after infection (Fig 8B). Taken together, these data, especially when combined with the earlier data on TLR3, indicate that nucleic acid receptors are much more important for the recognition of *L*. *pneumophila* than previously recognized.

### C-type lectin receptors are not required for the cytokine response of human macrophages to *L*. *pneumophila* infection

**Fig 9:**
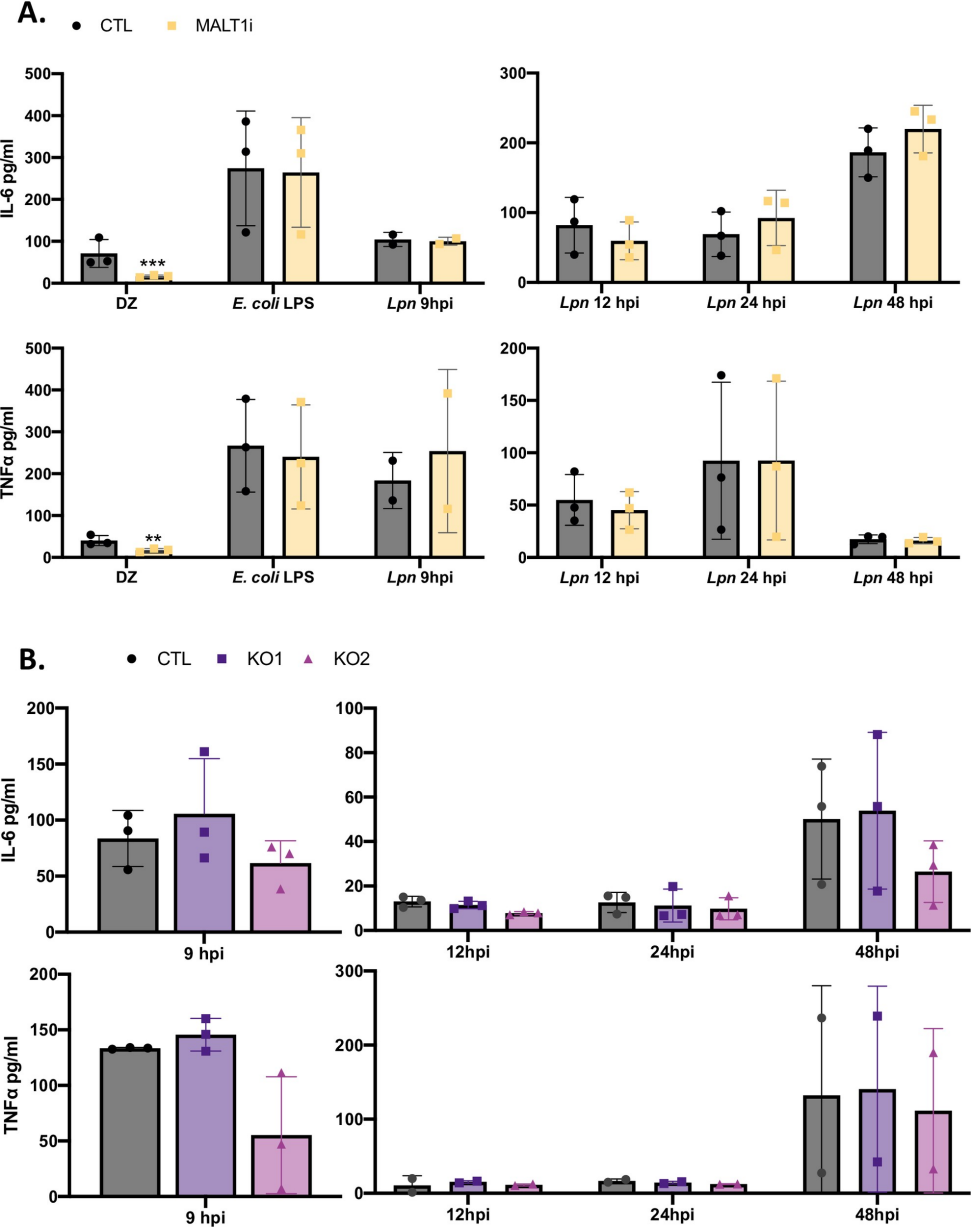
Assessing the requirement for C-type lectin receptors in the cytokine response of human macrophages to *L*. *pneumophila* infection. (A) U937 cells were pre-treated with either the MALT1 inhibitor Z-VRPR-FMK (MALT1i, yellow bars) or the vehicle control, DMSO (CTL, black bars). They were then exposed to either depleted zymosan (DZ) or *E*. *coli* LPS for 12 h (left panel), or infected for 9, 12, 24, or 48 h with strain 130b (*Lpn*) inoculated at a MOI 20 (for 9-h infection; left panel) or 0.5 (for 12, 24, and 48-h infections; right panel), and levels of secreted IL-6 (top) and TNFα (bottom) determined by ELISA. (B) U937 cells expressing a non-targeting CRISPR guide plasmid (CTL, black bars) and two independent clones of U937 cells containing a CRISPR/Cas9-generated MCL mutation (KO1, KO2; purple and magenta bars) were infected for 9, 12, 24, or 48 h with strain 130b (*Lpn*) inoculated at a MOI 20 (for 9-h infection; left panel) or 0.5 (for 12, 24, and 48-h infections; right panel), and levels of secreted IL-6 (top) and TNFα (bottom) determined by ELISA. Cytokine levels (pg/ml) secreted during infection were calculated relative to serial dilution of the recombinant cytokine standard. The graphs show the average cytokine levels (*n* = 3) pooled from three independent experiments, done in technical triplicate, with standard errors. Asterisks indicate points at which the values for samples from inhibited cells were significantly different from those for samples from CTL cells (**P < 0.01, ***P < 0.001, by Student’s *t* test).

Next, we considered the importance of surface C-type lectin receptors (CLRs) for *L*. *pneumophila* infection. Although these receptors are well-known for their importance in fungal infections, their role in bacterial infections has been minimally explored and in the case of *L*. *pneumophila* limited to several epidemiological studies [91–94]. The C-type lectin domain family-7 member A (Dectin-1), C-type lectin domain family-6 member A (Dectin-2), macrophage-inducible C-type lectin (Mincle), and C-type lectin domain family-SF member 8 (MCL, also known as CLECSF8) have been shown to respond to carbohydrate-derived PAMPs of bacteria [93, 95,96]. Based on studies using *Mycobacterium* spp., Dectin-1 recognizes β-glucans, Mincle responds to α-mannose, and Dectin-2 recognizes α-mannans and O-linked-mannobiose-rich glycoproteins [93]. On the other hand, MCL responds to trehalose 6,6’-dimycolate, based on experiments done with lung pathogens *Mycobacterium* spp. and *Klebsiella pneumoniae* [93,97]. Dectin-1, Dectin-2, and Mincle have a common downstream adapter called Mucosa-associated lymphoid tissue lymphoma translocation protein 1 (MALT1), and a chemical inhibitor of MALT1 (i.e., Z-VRPR-FMK) is commonly used to judge the role of these CLRs in various situations [98,99]. Hence, we examined the ability of Z-VRPR-FMK to alter the cytokine response of U937 cells upon *L*. *pneumophila* infection. As expected, the inhibitor decreased the response of the macrophages to depleted zymosan, a known agonist of multiple CLRs [95,100,101], but showed no off-target inhibition of the cell’s response to LPS (Fig 9A). The MALT1 inhibitor also did not affect the macrophage’s production of IL-6 and TNFα following *L*. *pneumophila* infection whether examined at the early 9-h time point (Fig 9A, left panels) or at 12, 24, and 48 h post infection (Fig 9A, right panels). CLR MCL is not upstream of MALT1 [93,95]. Therefore, we used CRISPR/Cas9 KO to probe for the role of MCL in *L*. *pneumophila* infection of U937 cells (S2 Table). Two independent MCL KO lines were not impaired in their ability to produce IL-6 and TNFα following *L*. *pneumophila* infection (Fig 9B). Taken together, these data indicated that CLRs are not required (major) PRRs in the response of human macrophages to *L*. *pneumophila* infection. However, it is possible that the CLRs are redundant with another PRR, including untested CLRs [93], involve the production of a cytokine not assayed here, and/or are only relevant at a different stage.

### The TLR4-signaling axis is required for cytokine response to *L*. *pneumophila* LPS

**Fig 10:**
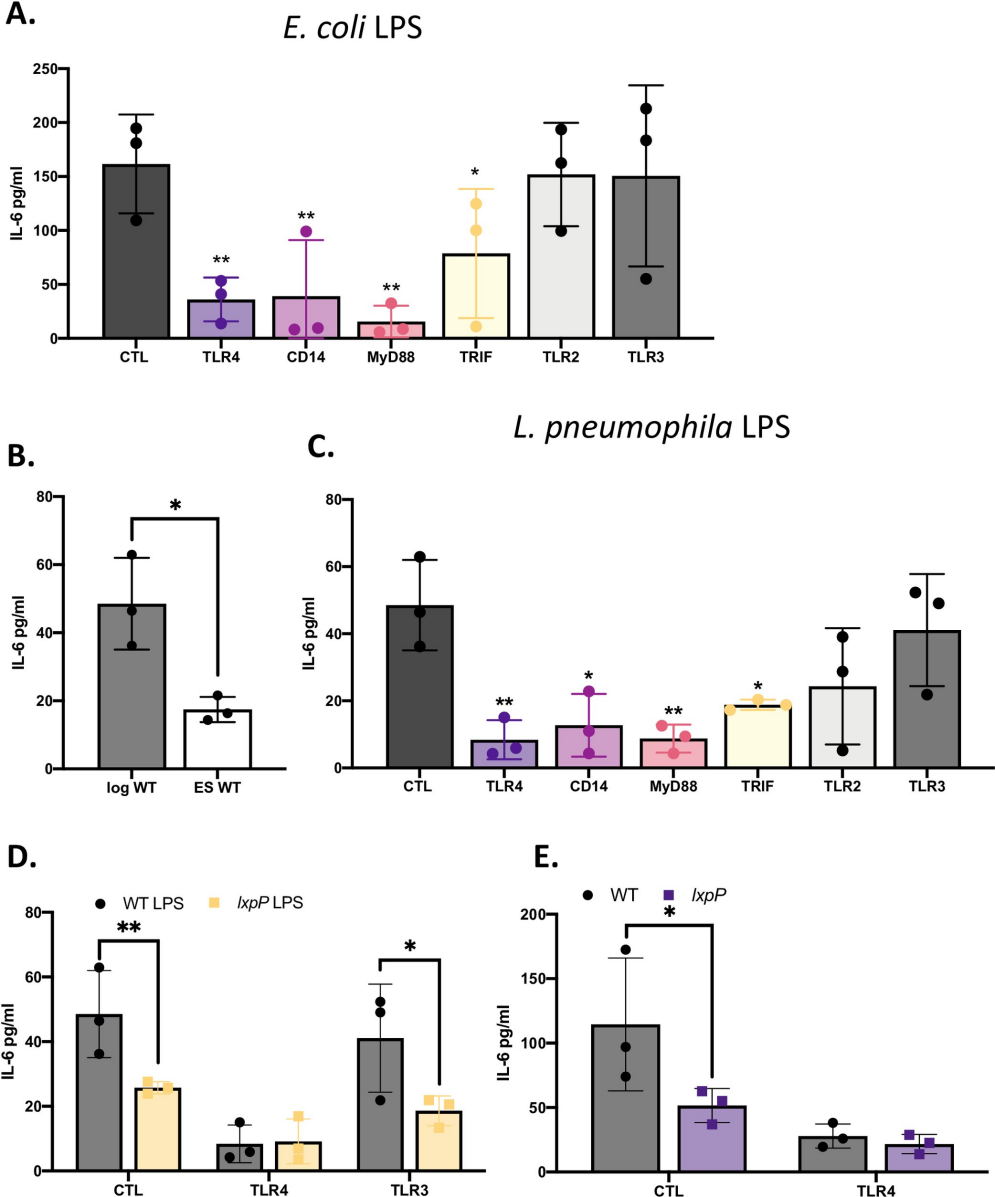
Effect of the TLR4 signaling pathway on the response of human macrophages to *L*. *pneumophila* wildtype and mutant LPS. (A) U937 macrophages expressing a non-targeting CRISPR guide plasmid (CTL) and U937 cells containing a CRISPR-generated KO of either TLR4, CD14, MyD88, TRIF, TLR2 or TLR3 were treated with *E*. *coli* LPS for 12 h and then levels of secreted IL-6 determined by ELISA. (B) CTL U937 cells were treated with *L*. *pneumophila* LPS purified from either log phase (Log) or early-stationary phase (ES) cultures of wildtype strain 130b (WT), and then secreted IL-6 levels determined as above. (C) CTL U937 cells and the various KO U937 cells noted in (A) were treated for 12 h with LPS obtained from ES cultures of strain 130b and then IL-6 determined by ELISA. (D—E) CTL, TLR3 KO, and/or TLR4 KO U937 cells were either exposed to LPS purified from ES WT or *lxpP* mutant bacteria for 12 h (D) or infected with WT or *lxpP* mutant bacteria for 9 h following inoculation with a MOI of 20 (E), and their levels of secreted IL-6 were then determined by ELISA. All graphs show the average cytokine levels (*n* = 3) pooled from three independent experiments, done in technical triplicate with standard errors. Asterisks indicate either the values for samples from KO cells that were significantly different from those for samples from CTL cells (A, C), the values for samples from the *lxpP* mutant that were different from those of the WT (D, E), or the significant difference between the samples from log vs. ES phase LPS (B) (*P < 0.05, **P < 0.01 Student’s *t* test).

With the documentation of a set of new PRRs relevant for *L*. *pneumophila* recognition by human macrophages, most notably TLR3, TLR4, TLR2, TLR5, and DNA-PK, we began work to identify the cognate *L*. *pneumophila* PAMPs. To that end, we hypothesized that *L*. *pneumophila* LPS is a critical PAMP that is recognized by human macrophages and specifically the one engaging human TLR4. Although LPS is widely known to be the PAMP recognized by TLR4 following infection by a range of Gram-negative pathogens [17,20,24], prior experimental assessments of *L*. *pneumophila* on this particular topic were murine-based and they had concluded that TLR2 is the receptor responding to *L*. *pneumophila* LPS [31,33,34,43,49]. Therefore, we used our newly-made panel of KO U937 cells to look directly at how *L*. *pneumophila* LPS is recognized by human macrophages. As a prelude, we confirmed the TLR4-CD14 dependent response of our U937 cells to *E*. *coli* LPS, a known agonist that is stimulatory toward macrophages and endotoxic in the *Limulus* amoebocyte lysate (LAL) assay (Figs 10A, and S9A and S9B). Consistent with earlier reports [102], *L*. *pneumophila* LPS, whether obtained from log or stationary phase cultures, was endotoxic in the LAL assay (S9A Fig). More to the focus of the present study, LPS obtained from log-phase *L*. *pneumophila* was more stimulatory for U937 cells, especially as measured by IL-6 production, than was an equivalent amount of LPS from stationary-phase cultures (Figs 10B and S9C), and hence LPS from log-phase legionellae was used for subsequent experiments. We observed that the TLR4 KO U937 cells showed a major reduction in IL-6 and TNFα production upon treatment with *L*. *pneumophila* LPS (Figs 10C and S9D). Supporting this finding, the response to *L*. *pneumophila* LPS was also diminished for macrophages lacking MyD88, TRIF, and the TLR4 co-receptor CD14 (Figs 10C and S9D). Compatible with these data, CD14 KO cells also had a diminished response to *L*. *pneumophila* infection itself (S9E Fig). But, the sensing of *L*. *pneumophila* LPS was not significantly lost in cells lacking TLR2 or TLR3 (Figs 10C and S9D), demonstrating specificity to the reactions we were observing. These data strongly suggested that human macrophages recognize *L*. *pneumophila* LPS through their CD14-TLR4 axis. Previously, we isolated a *lxpP* mutant of *L*. *pneumophila* strain 130b that lacks an acyltransferase which is predicted to influence fatty acids that are added to the lipid A moiety of LPS [103,104]. Because lipid A is critical for host recognition of LPS [105], we hypothesized that LPS from this mutant would be altered in its ability to trigger cytokines. Although the mutant’s purified LPS retained endotoxic activity (S9A Fig), it stimulated less IL-6 when compared to wildtype’s LPS, and the difference between the two was lost upon exposure to U937 cells lacking TLR4 but not TLR3 (Fig 10D). Moreover, when we then did infections, the *lxpP* mutant triggered less of a cytokine response than did the wild type, and this difference was lost upon exposure to TLR4 KO macrophages (Fig 10E). Finally, the mutant, unlike wild type, did not trigger a response from TLR4- transfected Hek-Blue cells (Fig 4C). Thus, there was a clear correlation between the relative ability of a *L*. *pneumophila* strain to trigger TLR4-dependent cytokines and the relative ability of its purified LPS to do the same. Together, our data demonstrate that not only is human TLR4 necessary and sufficient for sensing *Legionella* bacteria, but it is necessary for human cells to recognize *L*. *pneumophila* LPS.

## Discussion

The current study arguably represents the most comprehensive examination of the role of PRRs in the ability of human macrophages to recognize *L*. *pneumophila* infection. Most notably, we documented a critical role for TLR3, TLR4, and their adaptor TRIF in the ability of human macrophages to produce cytokines in response to *L*. *pneumophila* infection; such a role for TLR3 and TLR4 was overlooked in prior murine-based studies [26,28–32, 43,106]. Epidemiological studies had found that polymorphisms in the human TLR4 gene either predispose or restrict individuals from developing Legionnaires’ disease [41,45], and our results suggest that one explanation for this may be variations in the ability of macrophages to recognize infecting legionellae via TLR4. We further demonstrated that human TLR2, TLR5, and their adaptor MyD88 are also required for an optimal cytokine response; this result, unlike the one involving TLR3 and TLR4, does align with prior murine-based studies [26,29,31,33,43,49,51,56]. While examining these TLRs, we also confirmed that the adaptors TRAM and TRAF6 and the receptor CD14 are critical for the cytokine response of human macrophages to *L*. *pneumophila*. Although we did not find a required role for CLRs, we did gain evidence for cytosolic nucleic acid sensors cGAS-STING (and adaptor TBK1) and DNA-PK having roles in the human macrophage response to *L*. *pneumophila*.

We focused on the roles of PRRs during the initial phase of intracellular infection, as studies involving *L*. *pneumophila* and others have shown the importance of the early cytokine response for subsequent mobilization of other macrophages and neutrophils and the development of the adaptive immune response [107,108]. Indeed, we documented that the secreted factors influenced by TLR3, TLR4, and TRIF have at least one important consequence, namely the ability to induce the migration of human neutrophil-like cells across a type II epithelial cell layer of human lung origin. In our analysis, the cytokine linked most clearly linked to TLR3, TLR4, and TRIF was IL-6, and compatible with that finding, an epidemiological study of *L*. *pneumophila* infections reported that polymorphisms in human TLR4 correlate with alterations in IL-6 levels [45]. However, our chemical inhibitor studies and multiplex ELISA and RNA arrays indicated that other cytokines (TNFα, IL-1β, IL-10) and stimulating molecules (CSF2, CSF3, CXCL8, CXCL10) are also influenced by these PRRs. TRIF and MyDD88 proved to be important in the first 3 to 9 h after macrophages were infected, signaling that their associated TLRs are recognizing invading and initially-replicating legionellae. That TLR2 and TLR5 were critical confirmed the role of cell-surface TLRs that are associated with MyD88. That we also found a required role for TRIF-associated TLR3 highlights that there is recognition of *L*. *pneumophila* within its vacuolar compartment. The documented importance of TLR4, which is associated with MyD88 when on the surface, or TRIF when at an endosome, further suggests the relevance of both cell-surface and endosomal TLRs. Since CD14, a cell-surface co-receptor with TLR4, and TRAM, a second endosomal adaptor for TLR4, proved necessary for the full cytokine response, we infer that both surface and endosomal TLR4s are sensing the legionellae. Thus, human macrophage recognition of *L*. *pneumophila* through TLRs is both temporally and spatially regulated and encompasses at least four TLR pathways. Because the magnitude of the effect of the gene KO was comparable across these four TLRs, we posit that TLR2, TLR3, TLR4, and TLR5 are equally important in the macrophage response to *L*. *pneumophila* at least at the early stages of infection. We gained evidence that the/a *L*. *pneumophila* PAMP recognized by human TLR4 is LPS and strongly suspect, based on the literature, that the *L*. *pneumophila* PAMP sensed by human TLR3 is a nucleic acid; these topics will be further discussed momentarily. Given what is known regarding other bacteria interacting with macrophages, we posit that human TLR2 recognizes *L*. *pneumophila* lipoprotein(s) [109,110], and that the role of human TLR5 involves the sensing of *Legionella* flagellin [111]. Our finding that KO of TLR9 does not diminish the levels of IL-6 and TNFα indicates that not all (endosomal) TLRs participate in the response to *L*. *pneumophila*. On the other hand, it is possible that the role of TLR9 is redundant with another PRR, involves the production of a cytokine not assayed here, and/or is only evident at a different stage of infection.

**Fig 11:**
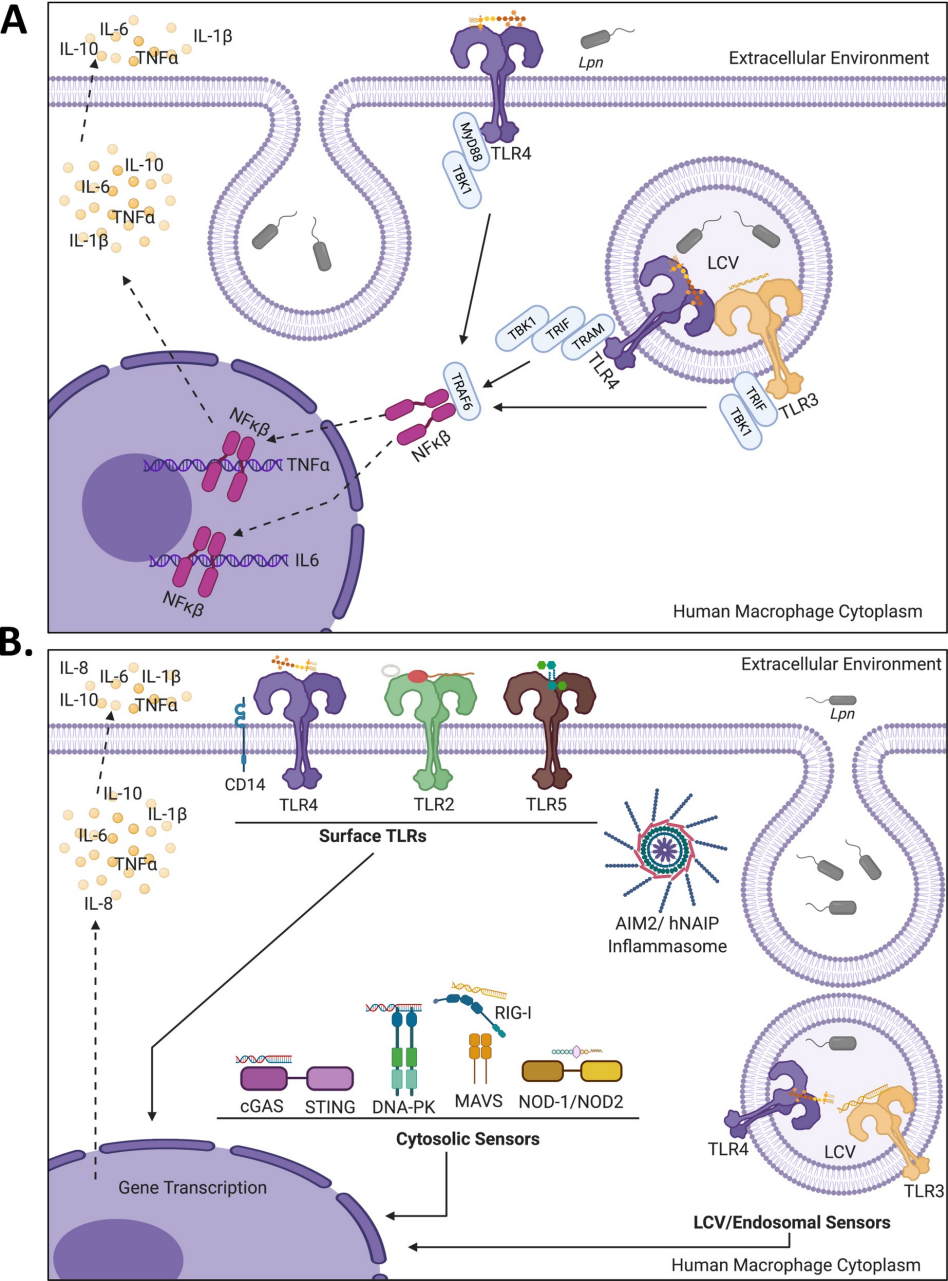
Summation of TLR3, TLR4, and other PPRs required for the human macrophage response to *L*. *pneumophila* infection. (A) The TLR3 and TLR4 signaling axes. TLR4 recognizes *L*. *pneumophila* (*Lpn*) LPS at both the macrophage surface and the endosomal / *Legionella*-containing vacuole (LCV) compartment. TLR4 at the surface signals via MyD88, whereas endosomal-localized TLR4 requires the TRIF/TRAM adaptors. TLR3 recognizes dsRNA presumably originating from the LCV and then utilizes the TRIF adaptor to begin signal transduction. TLR4/MyD88, TLR4/TRAM/TRIF, and TLR3/TRIF all engage downstream TBK1 and finally TRAF6 leading to transcription factor (e.g., NF-κΒ {shown}, AP-1) activation, which induces cytokine gene transcription (e.g., TNFα and IL-6) and its associated protein synthesis and secretion (dashed line arrows). In this study, we confirmed the TLR4- and TLR3-dependency of IL-6 and TNFα as well as IL-1β and IL-10. (B) Human PRRs that recognize and respond to *Lpn* infection, based on the results from the current study as well as past work (see main text for references). At the macrophage surface, TLR4 and its co-receptor CD14 work to recognize LPS, TLR2 likely senses a lipoprotein(s) and/or a component of outer membrane vesicles, and TLR5 detects flagellin. At the level of the endosome / LCV, TLR3 recognizes dsRNA, while TLR4 recognizes LPS. Within the macrophage cytosol, DNA-PK and cGAS/STING sense DNA, while RIG-I/MAVS recognizes dsRNA species, including those generated by the action of Pol III on released DNA, and NLR’s NOD1 and/or NOD2 recognize peptidoglycan. Finally, cytoplasmic inflammasomes, both AIM2 and hNAIP-dependent ones, recognize DNA, peptidoglycan, LPS, and flagellin. Regardless of the PRR or its location, signal transduction leads to the upregulation of cytokine gene transcription (solid black arrows). Cytokines that are known to be produced and secreted by human macrophages upon *L*. *pneumophila* infection include IL-1β, IL-6, IL-8, IL-10, and TNFα (dashed line arrows). PPRs that have been examined by KO analysis but were found not to be required for the production of cytokines (as measured by IL-6 and TNFα levels) include TLR9, MCL, and the Malt1-dependent CLRs. Diagrams were created with BioRender.com.

Through application of purified LPS to WT vs. TLR4 KO (and CD14 KO) macrophages, we documented that human TLR4 recognizes *L*. *pneumophila* LPS. In *L*. *pneumophila*, the O-antigen chain of LPS, which is named legionamnic acid, lacks free hydroxyl groups making it rather hydrophobic, and the lipid-A moiety is made of long-chain branched fatty acids that may explain the low endotoxicity of *L*. *pneumophila* LPS [103,112]. Typically, LPS is released from bacterial surfaces, including in outer-membrane vesicles [113]. This phenomenon occurs for *L*. *pneumophila*, both in log-phase cultures and in the LCV in macrophages [114,115]. Thus, we posit that LPS released from the *L*. *pneumophila* cell is engaging human TLR4, whether at the macrophage surface or at the level of the LCV. For three reasons, we posit that it is the lipid A of *L*. *pneumophila* that is engaging TLR4. First, when examined in other systems, the lipid-A moiety is the part that is recognized by TLR4 [103,116]. Second, we observed that infection with a *L*. *pneumophila* mutant bearing altered lipid A triggers a decreased, TLR4-dependent cytokine response, as did the LPS from that mutant. Third, four serogroups of *L*. *pneumophila*, which differ in their O-antigen [103], triggered a similar TLR4-dependent cytokine response, implying that this part of the LPS is not engaging TLR4. Though finding that *L*. *pneumophila* LPS is sensed by human TLR4 is not surprising when considering the many other bacteria that have been similarly characterized, it is a major change for the *L*. *pneumophila* field, since, as noted above, the long-standing view has been that *L*. *pneumophila* LPS is recognized by TLR2 and that TLR4 is not important, as determined from murine models [13,18,25,26,28,31,33,34,117]. While LPS is the canonical PAMP for TLR4, there are other TLR4-activating signals, including glycoinositolphospholipids, lipopeptidophosphoglycans, mannuronic acid, and endogenous danger-associated molecular patterns [24,110,116,118]. Thus, there might be another aspect of *L*. *pneumophila* that also engages human TLR4. Whether signaling from the surface or an endosome, TLR4 leads to the activation of factors such as NF-κΒ and AP-1 that result in the upregulation of genes including those for cytokines (Fig 11A).

TLR3 recognizes dsRNA and hence, is most often studied in context of viral infections [107]. Yet, in studies using C57BL/6 mice or human OE and HeLa cells, TLR3 has also been implicated in the recognition of bacterial pathogens, *Brucella abortus*, *Chlamydia muridarum*, and *Francisella tularensis*, and in the case of *B*. *abortus*, TLR3 was further implicated in the sensing of bacterial dsRNA [119,120]. In human cells, TLR3 resides in the endoplasmic reticulum (ER) [121], and upon stimulation with dsRNA, is trafficked to endocytic vacuoles. TLR3 signaling, which, as noted above, is dependent on binding and activation of TRIF, TRAF6, and TBK1, typically triggers activation of the NF-κΒ and AP-1 [20,107]. Early in its intracellular growth cycle (i.e., a time frame examined in our study), *L*. *pneumophila* recruits smooth vesicles from the ER to its endosomal home [16,17]. Therefore, we posit that TLR3 comes to reside at the LCV at which point it engages bacterial RNA species that are released into the lumen of the vesicle and then signals through its adaptors to upregulate cytokine genes (Fig 11A). Supporting this scenario, *L*. *pneumophila* is known to produce dsRNA (e.g., sRNA’s) during intracellular infection [122,123]. Given the fact that a small percentage (~3%) of wild-type *L*. *pneumophila* traffic to the phagolysosome during infection of human macrophages and monocytes [124,125] triggering of TLR3 may also result from dsRNA species that are released upon lysis of those bacteria that are trafficked to the degradative phagolysosome.

From our analysis of KD/KOs of cGAS-STING and DNA-PK, coupled with results from an earlier study of ours and that of another group [22,86], it is clear that nucleic acid-sensing within the cytoplasm is a major component of the human macrophage response to *L*. *pneumophila*. This represents the latest addition to a growing list of bacterial pathogens that are sensed by cGAS-STING that includes *B*. *abortus*, *F*. *tularensis*, *L*. *monocytogenes*, and *Mycobacterium tuberculosis*, among others [126]. The finding concerning DNA-PK is more novel, since there are only a few prior examples, including the recognition of *Escherichia coli* DNA and DNA damage resulting from infection with *L*. *monocytogenes* [127,128]. It has been proposed that *L*. *pneumophila* PAMPs might enter the host cell cytoplasm through a semi-permeable LCV membrane or by active secretion via the bacterium’s type IV secretion system [5,70]. Thus, we posit that the delivery of *Legionella* nucleic acid is occurring through one of these means.

It is worthwhile to contemplate why TLR3 AND TLR4 had been missed as being important in earlier murine-based studies. Prior to the current study, the interaction of TLR3 with *Legionella* had only been studied in a C57BL/6 mouse, where the authors did not find any effect of a TLR3 KO on the survival of *L*. *pneumophila* (strain JR32) in the lungs or the production of IL-1α in the serum [27]. More numerous, murine-based studies of TLR4, which made use of either TLR4 KO mice in the BALB/c, A/J, and C57BL6/J backgrounds, TLR4 mutant C3H/H3J mice, or explanted macrophages from the TLR4 KO animals, also did not observe any reductions in intrapulmonary growth or whole-body inflammatory responses, including the production of TNFα and IL-6 [26–31,34,43,49,129]. Many of these studies utilized flagellin mutants, since *Legionella* flagellin is recognized by the murine Naip5 inflammasome, leading to blocks in the ability of the bacteria to replicate intracellularly in most murine systems, save the A/J mice [130]. When we performed KD of TLR3 and TLR4 in both A/J BMDMs and murine PBMC-derived macrophages, there was no loss in the cytokine response to wild type *L*. *pneumophila*. Therefore, we hypothesize that, during *L*. *pneumophila* infection of murine macrophages, TLR3 and TLR4 are absent from the microenvironment containing the bacterium, the requisite PAMPs are not produced or delivered, and/or the murine TLRs simply do not bind the present PAMP. Pertinent to the last scenario, murine and human TLRs do differ significantly in the sequence of their antigen binding domains, and therefore they can differ in their ability to bind like PAMPS [116,118]. Indeed, this might explain why past studies implicated TLR2 (not TLR4) as the murine PRR for *L*. *pneumophila* LPS [26,31,33]. In support of there being a possible difference in the delivery or spread of PAMPs, we previously observed that some secreted proteins of *L*. *pneumophila* traffic out of the LCV in human macrophages but not out of LCV in murine macrophages [70]. Our current findings represent one in a growing list of examples showing differences between the murine vs. human immune response to *L*. *pneumophila* infection. Several recent reports indicate that signaling through caspase-1, caspase-7, and interferon priming all appear to differ between human and murine *Legionella*-infected cells [2,70]. Taken together, these observations indicate that murine models of *L*. *pneumophila* infection do not sufficiently represent human infection, and more human-based work should be done in order to better understand important aspects of the immune response to *L*. *pneumophila*.

In summary, our comprehensive analysis of PRRs, when combined with past human-based studies [2,22,106,117,131–137], provides a much-revised view of the human macrophage response to pathogenic *Legionella* (Fig 11B). The continued characterization of how human macrophage PRRs and their associated pathways engage with *L*. *pneumophila* also has the potential to reveal new therapeutic strategies for dealing with Legionnaires’ disease. Finally, our observations, especially those concerning the understudied nucleic acid sensors, have implications for investigating the human innate immune response to a range of other bacterial pathogens.

## Materials and methods

### Ethics statement

Experiments using mice (i.e., explanted macrophages) were approved by the Institutional Animal Care and Use Committee (IACUC) at Northwestern University under protocol number IS00009832.

### Bacterial strains, bacteriological media, and chemicals

*L*. *pneumophila* clinical isolate strain 130b (American Type Culture Collection {ATCC} BAA-74) was used as the wildtype strain for nearly all experiments [22]. Strain NU360, a *lpxP* mutant of strain 130b that is defective for modification to lipid A, was previously described [104]. Other wildtype strains of *L*. *pneumophila* tested for intracellular growth and/or cytokine elicitation were clinical isolates Philadelphia-1 (ATCC 33152), Paris (ATCC 700174), Chicago-8 (ATCC 33823), 82A3105 (ATCC 43736), and 1169-MN-H (ATCC 43703) [138,139]. Other species of *Legionella* that were tested included *Legionella birminghamensis* strain 1407-AL-H (ATCC 700709), *Legionella longbeachae* strain Long Beach 4 (ATCC 33462), and *Legionella micdadei* Stanford-R [104,139,140]. Prior to infections, all *L*. *pneumophila* strains and other *Legionella* species were cultured for 3 days at 37°C on buffered charcoal yeast extract (BCYE) agar [141]. In order to obtain LPS samples, *L*. *pneumophila* strain 130b and its mutant were grown with shaking at 37°C in buffered yeast extract (BYE) broth, with the stage of bacterial growth determined by measuring the optical density (OD) of the cultures at 660 nm using a DU720 spectrophotometer (Beckman) [142,143]. Unless noted otherwise, chemicals were purchased from Sigma-Aldrich.

### Mammalian cell cultures

In order to examine *L*. *pneumophila* intracellular infection and its associated cytokine response, a variety of established *in vitro* and *ex vivo* mammalian cell preparations were utilized. Specifically, this included the human U937 cell line (ATCC CRL-1593.2), human HEK-293 cell line (ATCC CRL-11268), human A549 cell line (ATCC CCL-185), human HL-60 cell line (ATCC CCL-240), human primary macrophages derived from peripheral blood mononuclear cells (PBMC) (ATCC, #PCS-800-011), and murine primary macrophages derived from either the PBMCs or bone marrow (BMDMs) obtained from 6- to 8-week-old female A/J mice (Jackson Laboratory). Euthanasia of mice was achieved by carbon dioxide inhalation followed by cervical dislocation. The U937, A549 and HL60 cell lines were maintained in RPMI 1640 medium (catalog number 100–40; Corning) supplemented with 10% fetal bovine serum (FBS) (catalog number S11150; Atlanta Biologicals) and 1% Penicillin-Streptomycin (PS), as previously described [22,144]. Hek293 cells were cultured according to ATCC protocols in DMEM medium (catalog number 10-017CV; Corning) containing 10% FBS and 1% PS. The U937 cells were differentiated into a macrophage-like state by culturing them in the presence of phorbol 12-myristate 13-acetate (PMA) [22]. HL-60 cells were differentiated into a neutrophil-like state by culturing them in the presence of dimethyl sulphoxide (DMSO) [144]. Murine BMDMs were collected and differentiated into a macrophage-like state as before [22]. Murine PBMCs were isolated as previously described [145]. PBMCs, whether from mice or human blood, were centrifuged at 100 x *g* for 10 min, washed twice in phosphate buffered saline (PBS) (Corning, #21–040;) containing 10% FBS, and then the mononuclear fraction was resuspended in RPMI medium containing 15% FBS and placed into 10-cm polystyrene wells (Falcon, #CLS430591). Following incubation for 2 h at 37°C, non-adherent cells were excluded from the plate by washing three times. The remaining adherent mononuclear cells were differentiated to a macrophage-like state by further incubation in 10% FBS, 1% PS RPMI medium containing 50 ng/ml of murine MCS-F (R & D, #416-ML-050/CF) or human MCS-F (R & D, #215-MC-005) differentiating factor for 6 days, with one media change, as previously described [146].

### Gene knockdown (KD) in human macrophages

In order to identify pathways that are important for the ability of human macrophages to sense *L*. *pneumophila*, knockdowns in gene expression were done using either shRNA or siRNA. As previously described [22], stable U937 KD cell lines were generated by transduction with a vesicular stomatitis virus glycoprotein (VSV-G)-pseudotyped lentivirus that encoded an shRNA molecule targeting the gene of interest. As a control, a nontargeting shRNA plasmid (scramble) was separately introduced into the U937 cells [22]. The sources used for shRNA are listed in S1 Table. For KD in PBMC-derived macrophages, a cell type that is not easily stably transduced, VSV-G siRNA (Dharmacon) and the non-targeting control (S1 Table) were transiently transduced using a nucleofector kit according to the manufacturer’s guidelines (Lonza, #VPA-1009). In order to quantify the shRNA and siRNA KD’s, U937 cell or PBMC-macrophage mRNA was collected and qPCR analysis performed as previously described [22]. Briefly, 1 X 10^7^ KD or CTL cells were collected and lysed using 1 ml TRIZOL reagent (Invitrogen, #15596018). mRNA was reverse transcribed into cDNA using SuperScript IV Reverse Transcriptase (Invitrogen, #18090010) following the manufacturer’s protocol. After quantification using a NanoDrop spectrophotometer (Thermo Fisher), the cDNA was amplified by qRT-PCR using SYBR Green (Bio-Rad, #1708882) and the primers listed in S3 Table. CFX RT-PCR software (Bio-Rad, #1708884) was used to calculate cycle threshold (CT) values for the genes of interest by normalizing to the housekeeping gene, β2 microglobulin (S3 Table), and the fold-induction relative to control was calculated [22]. Immunoblot analyses were further used to confirm the knockdown of target proteins from siRNA and shRNA techniques [22].

### Gene knockout (KO) in human macrophages

In order to assess which PRR pathways were important for the ability of human macrophages to sense *L*. *pneumophila*, a knockout system using CRISPR-Cas9 was performed as previously described [147–150]. Briefly, HEK239T cells were transfected using FuGene transfection reagent (Promega, #E2331) with third-generation packaging plasmids pVSV-G and PsPAX2 (Addgene) and lentiCas9-Blast plasmid (Addgene). Virus was harvested 72 h later (0.45-μm filter) and used to transduce non-PMA treated U937 cells [148] allowing the Cas9 gene to stability integrate into the genome. After single-cell clonal selection with blastacydin (Thermo Fisher) and confirmation that the Cas9 protein was now expressed, the U937 cells were individually transduced using lentiviral produced pLentiGuide-puro plasmids (Addgene) as above. Guide Plasmids were designed following previously described protocols [147,149,150] and were created using Esp3I (BSMB1) restriction sites and the Brunello list [147] of Cas9 gene specific targeting library (S2 Table). Guide plasmid targeting AASV1 (AA) gene, a non-coding section of the human genome, was used as a negative control for all later experiments. Guide plasmid targeting PVSG1, coding for a chaperone protein, was used as a confirmation of gene editing–i.e. quantification of cell death. After successful transduction of guide plasmids, single-cell cloning was utilized to obtain mutants carrying a clonal mutation, which were confirmed for the presence of a Cas9-generated deletion in the target gene. Primers spanning the protospacer adjacent motif (PAM) region were designed, and clonal cell populations were PCR-amplified (S4 Table), gel purified (Promega kit), and sequenced (Eurofins) in order to confirm the KO. Sequences of KO targets were compared to AA control (CTL) and analyzed for deletions and modifications around the PAM region. Clones with larger (>10-bp) deletions were utilized for infection experimentation. In the case of TRIF, MyD88, the TLRs, TRAF6, CD14, and MCL, two individually derived clones (using different gene regions) were obtained by the above-described method and utilized in infection and cytokine assays (S2 Table). A single clone was also derived for the analysis of TRAM (S2 Table). For DNA-PK U937 CRISPR/Cas9 KO’s, validated cell lines were purchased from Synthego (S2 Table). Sequencing was done to confirm the mutations.

### Intracellular infection of and cytokine production by macrophages

*L*. *pneumophila* infection of the various macrophages (specified above) was done according to published methods [22,143,151]. Briefly, monolayers of macrophages were plated in RPMI medium at a concentration of 1 x 10^6^ per well in 24-well plate (Corning, #CLS3527 tissue culture-treated), exposed to bacteria at a MOI of 0.5, 5, 10, 20, or 40 (as indicated) for 2 h in order to allow bacterial entry, and then washed three times with RPMI medium to remove remaining extracellular legionellae. The tissue culture medium was replenished as RPMI medium with 10% FBS, and *L*. *pneumophila* intracellular infection was allowed to proceed for up to 48 h. To judge bacterial intracellular growth, the monolayers were lysed at the indicated time points, and colony forming units (CFU) determined by plating on BCYE agar. In order to monitor cytokine expression by the macrophages, tissue culture supernatants or mRNA were collected at the indicated time points, frozen at -80°C, and analyzed by ELISA or RT-PCR array, as described in the next section. In some trials, chemical inhibitors were used to block a particular host cell recognition pathway as an alternative means toward judging the impact of that pathway on *L*. *pneumophila* infection. For the inhibition of TLR3, Cu CPT 4a (TOCRIS, #4883) was used [63], for TLR4 inhibition, TAK-242 (TOCRIS, #6587) [64], and for MALT1 blockade, Z-VRPR-FMK (TOCRIS, #4645) [93,98,99]. Macrophages were pretreated with the inhibitor (i.e., 30 μM for Cu CPT 4a, 15 μM for TAK 242, or 100 μM for Z-VRPR-FMK), or its vehicle control (0.1% DMSO in RPMI medium) for 1 h and then infected with *L*. *pneumophila* (as noted above) in the continued presence of inhibitor or vehicle control and secreted cytokine levels determined by ELISA at the indicated time points. As controls for confirming the correct action of the inhibitors the macrophages were also separately exposed to either 15 μg/ml of TLR3 agonist Poly (I:C) (Sigma-Aldrich, #P9582), 1 ng/ml of TLR4 agonist *E*. *coli* LPS, (eBioscience, #00-4976-93), or 50 μg/ml of MALT1 agonist DZ (InvivoGen, #tlrl-zyd) [17,20,24,65,98,99]. On-target effects were determined by a dose escalation study where the minimum inhibitory concentration was determined; i.e., the lowest concentration where the inhibitor blocked agonist-dependent signaling after treatment [63,64]. Off-target effects were determined during the dose escalation study using agonists of pathways not targeted by inhibitors, and validating that native signaling is not blocked by the inhibitor [63]. To that end, we employed *S*. *typhimurium* flagellin (InvivoGen, #tlrl-flic-50), a canonical agonist of TLR5, administered at 5 μg/ml [63]. In addition to control treatments involving the above-noted Poly I:C (15 μg/ml), *E*. *coli* LPS (1 ng/ml), and flagellin (5 ug/ml), CRISPR/Cas9 KOs and shRNA KDs were also examined for ON/OFF target effects by treatment with the TLR2 agonist PAM (InvivoGen, #vac-pms), administered at 50 ng/ml, and DNA PRR (TLR9, DNA-PK) agonists CpG (InvivoGen, #tlrl-dsl03) administered at 5μM, and cGAS/STING agonist [152–155] G3-YSD (InvivoGen tlrl-ydna), transfected at a concentration of 1 μg/ml.

### ELISA and RT-PCR array

To quantify cytokines secreted by macrophages after infection with *L*. *pneumophila*, tissue culture supernatants obtained at the indicated time points were analyzed by ELISA as previously described [22]. Following manufacturer’s recommended protocols, single ELISA plates were used to assay either human IL-6 (eBioscience, #88-70066-77), mouse IL-6 (Thermo Fisher, #88-7064-77), human TNFα (eBioscience, #88-7346-88), or mouse TNFα (eBioscience, #88-7324-22), with the quantification of cytokine levels determined using a plate reader (Biotek Synergy H1). The supernatants from human macrophages were also applied to a multiplex ELISA plate (Quansy Biosciences, #110933HU) and assayed according to manufacturer’s protocol [156]. This 16-plex plate was read using Q-View imager LS (Quansy Biosciences), and analyzed using Q-View software following the manufacturer’s guidelines. The RT^2^ Profiler PCR-Array (Qiagen, #PAHS-018Z) was used to determine the levels of cytokine gene expression in U937 cells following 1 h of infection with *L*. *pneumophila*. Following the manufacturer’s protocol, mRNA was isolated using a RNAeasy mini-kit (Qiagen, #74104) and then converted to cDNA using the First-Strand Kit (Qiagen, #330401). Software provided by the manufacturer was then used to calculate the fold-changes in gene expression.

### Transwell Co-Culture Assay

In order to detect chemotactic factors secreted by macrophages upon *L*. *pneumophila* infection, tissue culture supernatants were assayed for their ability to stimulate the movement of neutrophil-like human HL-60 cells across an A549 epithelial cell monolayer, as previously described [71,72]. HL-60 cells were differentiated to the neutrophil-like state by incubation in 100 ml of RPMI medium containing 1.3% DMSO and 10% FBS for 5 days [144]. To prepare the A549 cell layer, 3-μm, collagen-coated transwell inserts (Corning, #CLS3496-24EA) were incubated in RPMI medium with 10% FBS and 1% PS for 30 min, and then 1 x 10^5^ A549 cells were seeded onto each transwell and allowed to adhere for 48 h. The transwells containing an A549 cell layer were placed into 600 μl of either supernatant from infected U937 cells or RPMI medium alone. The differentiated HL-60 cells (above) were resuspended in RPMI medium to 5 x 10^5^ per 100 μl, and 100 μl of the suspension were applied to the top of the transwells. After 1 h of incubation, the number of differentiated HL-60 cells that had migrated through the A549 cell layer and into the lower chamber of the transwell were quantified using a Cell-Scepter counter (EMD Millipore). Control wells containing no HL-60 cells, or HL-60 cells with no transwells were used as negative and positive controls, respectively.

### Assays using Hek-Blue TLR3 and Hek-Blue TLR4 reporter cells

TLR3 / NF-κB / SEAP reporter HEK293 cells (InvivoGen, #hkb-htlr3) and TLR4 / NF-κB / SEAP reporter HEK293 cells (InvivoGen, #hkb-htlr4), along with corresponding null cell lines Null1 (InvivoGen, #hkb-null1) and Null2 (InvivoGen, #hkb-null2), were used to determine if human TLR3 and TLR4 can sense infection with *L*. *pneumophila*. In the reporter cells, an NF-kB-regulated promotor, whose expression is controlled by the levels of TLR activation, is fused to a secreted alkaline phosphatase (SEAP) reporter gene. SEAP hydrolyzes a colorogenic substrate in the assay’s detection media; thus, increases in SEAP expression lead to the production of a blue color [60,62]. The cell lines were cultured in DMEM containing 10% FBS and supplemented with antibiotics as supplied and specified by the manufacturer (InvivoGen). Hek-Blue cells were counted using a cell scepter cell counter (EMD Millipore), suspended in Hek-Blue Detection media (InvivoGen, #rep-qb), and then added to a 96-well plate (Corning, #353072) at a concentration of 1 x 10^5^ per well. Cells were either infected with *L*. *pneumophila* at a MOI of 50 or treated with the canonical agonists Poly (I:C) at 15 μg/ml or *E*. *coli* LPS at 1 ng/ml. Following 9 h of incubation, the SEAP activity was quantified by reading the absorbance at 635 nm. Results from the TLR3- and TLR4-transfected Hek-Blue cell lines absorbances were normalized to those of the null background cell lines and reported as relative absorbance units [59,60,62].

### Isolation and examination of *L*. *pneumophila* LPS

LPS was purified according to established protocols [102,112,157,158]. Briefly, *L*. *pneumophila* strains were grown at 37°C with shaking in 20 ml of BYE broth in a 120-ml flask to log-phase (i.e., ~OD_660_ = 1) or early stationary phase (i.e., ~OD_660_ 2.5). 5 ml of bacterial culture were centrifuged, and the resultant pellet was freeze-dried using vacuum freezing. The pellet was resuspended and vortexed in 90% phenol—10% endotoxin-free water at a concentration of 10 mg/ml, and then the suspension incubated at 65°C for 1 h, cooled on ice for 5 min, and finally centrifuged 10,000 × *g* at 4°C for 30 min. After collection, the aqueous phase was dialyzed at 4°C against Milli-Q (Millipore)-purified water using Slide-A-Lyzer Dialysis Cassettes with a molecular-weight cut-off of 3.5 kDa (Invitrogen). Following lyophilization of the sample, the resulting pellet was resuspended at a concentration of 10 mg/ml in endotoxin-free water containing 100 μg/ml DNase (EMD Millipore, #260913-10MU) and 25 μg/ml RNase A (Life Technologies, #AM2272) and statically incubated at 37°C for 1 h. Following the addition of 100 μg/ml of Proteinase K (Fisher Scientific, #25-530-049), the sample was further incubated for 1 h at 37°C. Hot phenol extraction was performed, and a subsequent dialysis done, as described above. Following lyophilization, the final pellet was resuspended at 10 mg/ml in endotoxin-free water. The concentration and endotoxicity of the purified LPS was confirmed using the LAL-assay (Thermo Fisher, #88282), as previously described [102,159]. To gauge the ability of LPS to trigger cytokine production, *L*. *pneumophila* LPS or *E*. *coli* LPS (control, as noted above) was added at concentrations of 100 ng/μl or 1 ng/μl, respectively, to wildtype and KO U937 cells, and then after 12 h of exposure, the levels of cytokine production were measured by ELISA as described above.

## Supporting information

S1 Fig qRT-PCR validation of the shRNA KD of TRIF and *L*. *pneumophila* dependent macrophage response determined by multiplex cytokine and mRNA analysis. (A) qRT-PCR of the TRIF gene in non-transfected (CTL), scramble-transfected (SCM), and TRIF KD U937 cells was used to test KD efficiency. The value for the CTL was set to 1, and the values for the others is represented as fold-change compared to CTL. (B) U937 cells were either not infected (UI) or infected with strain 130b at a MOI of 20, and the levels of sixteen different cytokines in culture supernatants at 9 h post infection were then ascertained by multiplex ELISA analysis, with the pg/ml levels presented using the indicated color scheme. Multiplex represents the analysis of three pooled biological replicates (*n* = 3) each done in technical triplicate. (C) U937 cells were either not infected or infected with strain 130b at a MOI of 20, and the levels of cytokine and chemokine transcripts at 1 h post-infection were then determined using a Qiagen RT-PCR Array. Fold-changes in transcripts were calculated relative to uninfected U937 cells and are presented in log10 units using the indicated color scheme. All ten factors indicated here had a significant increase in expression upon infection (*P* < 0.05, by Student’s *t* test). Asterisks indicate five cytokines whose levels were significantly greater in the infected monolayers (***P < 0.001, by Student’s *t* test).
(TIF)

S2 Fig Growth of *L*. *pneumophila* in U937 human macrophages with shRNA KD or CRISPR/Cas9 KO of TRIF and MyD88. (A–B) Non-transfected U937 cells (CTL) or U937 cells containing either an shRNA targeting TRIF (TRIF KD) or a non-targeting scramble shRNA (SCM) were infected with strain 130b (A) or Philadelphia-1 (B) at a MOI of 0.5, and then bacterial CFU were determined by plating at 0, 24, and 48 h post-infection (HPI). (C) U937 cell macrophages containing either a mutation in the TRIF gene (TRIF KO1, TRIF KO2) or MyD88 gene (MyD88 KO1, MyD88 KO2) or a non-targeting CRISPR Guide plasmid (CTL) were infected with strain 130b at a MOI of 20 and CFU determined as above. Data represent the mean CFU (on log_10_ scale) and standard error for triplicate samples and for each panel are representative of three independent experiments.
(TIF)

S3 Fig Validation of siRNA KD of TRIF, and shRNA KD of cGAS/STING. (A) Human PBMC-derived macrophages containing either an siRNA targeting TRIF (TRIF KD) or a non-targeting scramble siRNA (SCM) were examined by immunoblot for the presence of TRIF protein or GAPDH. The KD was done in technical duplicate and then assayed on two occasions. The mean fluorescent intensity quantification of the western blot was then used to determine the specific loss of TRIF. (B) U937 macrophages containing either an shRNA targeting cGAS (cGAS KD), STING (STING KD) or a non-targeting scramble shRNA (SCM) were examined by immunoblot for the presence of cGAS/STING protein or GAPDH. The KD was done in technical triplicate and then assayed on two occasions. The mean fluorescent intensity quantification of the western blot was then used to determine the specific loss of cGAS/STING was used to test KD efficiency. In (A-B), the value for the SCM was set to 1, and values for the others is represented as fold-change compared to CTL. Asterisks indicate points at which the values for samples from KD cells were significantly different from those for samples from SCM cells (* *P* < 0.05, ***P < 0.001, by Student’s *t* test).
(TIF)

S4 Fig Raw values for the multiplex cytokine analysis of TRIF and MyD88 KOs in Fig 2B. Control, TRIF KO, and MyD88 KO U937 cells were infected with *L*. *pneumophila* strain 130b at a MOI of 20, and the secreted levels of IL-1β, IL-6, IL-8, IL-10 and TNFα (as indicated on left side) at 0, 1, 3, 6, 9, 12, 24, and 48 h post infection (right side) were ascertained by multiplex cytokine ELISA, with pg/ml levels presented using the indicated color scheme (far right side). The multiplex system represents analysis of three pooled biological replicates (*n = 3*) each done in technical triplicate.
(TIF)

S5 Fig Quantification of ON/OFF target effects from shRNA KD and CRISPR/Cas9 KO in U937 macrophages. U937 cells expressing a non-targeting CRISPR guide plasmid (CTL, black bars) and U937 cells containing a CRISPR/Cas9-generated mutation (KO1) in either TLR3 (A), TLR4 (B), TRIF (C) and TLR2 (D) were either untreated (UT) or treated with Poly I:C, *E*. *coli* LPS, PAM, flagellin, or CpG, and the levels of secreted IL-6 and TNFα at 12 h post treatment were then determined by ELISA. Secreted cytokine levels (pg/ml) were calculated relative to serial dilution of the recombinant cytokine standard. Graphs show the average cytokine levels pooled from two independent experiments, done in technical triplicate, with standard errors. For ease of comparing the effects of the different treatments on a given KD/KO, the treated CTL result was presented in each row. Asterisks indicate points at which the values for samples from the KO/KD cells were significantly different from those for samples from CTL cells (*P < 0.05, **P < 0.01, ***P < 0.001, by Student’s *t* test).
(TIF)

S6 Fig Quantification of ON/OFF target effects from shRNA KD and CRISPR/Cas9 KO in U937 macrophages. U937 cells expressing a non-targeting CRISPR guide plasmid (CTL, black bars) and U937 cells containing a CRISPR/Cas9-generated mutation (KO1) in either TLR5 (A), TLR9 (B), cGAS (C), or DNA-PK (D) were either untreated (UT) or treated with Poly I:C, *E*. *coli* LPS, PAM, flagellin, CpG, or G3-YSD, and the levels of secreted IL-6 and TNFα at 12 h post treatment were then determined by ELISA. Secreted cytokine levels (pg/ml) were calculated relative to serial dilution of the recombinant cytokine standard. Graphs show the average cytokine levels pooled from two independent experiments, done in technical triplicate, with standard errors. For ease of comparing the effects of the different treatments on a given KD/KO, the treated CTL result was presented in each row. Asterisks indicate points at which the values for samples from the KO/KD cells were significantly different from those for samples from CTL cells (*P < 0.05, **P < 0.01, ***P < 0.001, by Student’s *t* test).
(TIF)

S7 Fig Requirement of TLR3 and TLR4 for the cytokine response of human macrophages to infection with *L*. *pneumophila* at different MOIs. U937 cells expressing a non-targeting CRISPR guide plasmid (CTL, black bars) and U937 cells with a CRISPR-generated KO of TLR3 or TLR4 (KO, purple and magenta bars) were either not infected (UI) or infected with *L*. *pneumophila* at a MOI of 5, 10, or 40 as indicated, and the levels of secreted IL-6 and TNFα at 9 h post-infection were then determined by ELISA. The cytokine levels (pg/ml) were calculated relative to serial dilution of recombinant cytokine controls. Graphs show the average cytokine levels (*n* = 2) pooled from two independent experiments, done in technical triplicate, with standard errors. Asterisks indicate points at which the values for samples from KO cells were significantly different from those of CTL cells (*P < 0.05, **P < 0.01, ***P < 0.001, by Student’s *t* test).
(TIF)

S8 Fig Requirement of TLR3 and TLR4 for the cytokine response of human macrophages to infection with different *L*. *pneumophila* serogroups and *Legionella* species. U937 cells expressing a non-targeting CRISPR guide plasmid (CTL, black bars) and U937 cells with a CRISPR-generated KO of TLR3 or TLR4 (KO, purple and magenta bars) were infected with *L*. *pneumophila* serogroup (SG) 1 strains Philadelphia-1 and Paris (A), *L*. *pneumophila* strains representing SG 7, 13, and 14 (B), and strains representing the *Legionella* species *L*. *longbeachae*, *L*. *birminghamensis*, and *L*. *micdadei* (C) at a MOI of 20, and the levels of secreted IL-6 at 9 h post-infection were then determined by ELISA. The cytokine levels (pg/ml) were calculated relative to serial dilution of recombinant cytokine controls. Graphs show the average cytokine levels (*n* = 3) pooled from three independent experiments, done in technical triplicate, with standard errors. Asterisks indicate points at which the values for samples from KO cells were significantly different from those of CTL cells (*P < 0.05, **P < 0.01, ***P < 0.001, by Student’s *t* test).
(TIF)

S9 Fig Assessing the requirement of the TLR4 signaling pathway for the cytokine response to *L*. *pneumophila* LPS, including raw values for the cytokine data in Fig 11. (A) LPS purified from 5-ml samples of log-phage (log) or early-stationary phase (ES) cultures of either wildtype *L*. *pneumophila* strain 130b (WT) or a *lpxP* mutant derivative of strain 130b (*lpxP*) were examined using the LAL-assay. The diluent used to resuspend the LPS was used as a negative control and also tested. The concentrations of LPS in each of the *L*. *pneumophila* samples (in technical duplicate) were then determined by comparison to a standard curve generated using the results obtained with the *E*. *coli* LPS. LPS concentrations are expressed as endotoxin units (EU) per ml, where one EU equals 0.1 to 0.3 ng LPS/ml, as per the manufacturer’s protocol. As depicted here, the LPS in the *L*. *pneumophila* samples corresponded to approx. 1–2 ng of *E*. *coli* LPS. Based on these data, all subsequent experiments utilized a defined amount of LPS. (B) CTL U937 cells were treated for 12 h with ~100 ng/μl of *L*. *pneumophila* LPS purified from either log phase (log) or early-stationary phase (ES) cultures of wildtype strain 130b (WT), and then secreted TNFα levels determined by ELISA. (C—D) U937 macrophages expressing a non-targeting CRISPR guide plasmid (CTL) and CRISPR-generated U937 KOs lacking either TLR4, CD14, MyD88, TRIF, TLR2 and TLR3 were treated for 12 h with either *E*. *coli* LPS at 1 ng/μl (C), or log-phase *L*. *pneumophila* LPS at 100 ng/μl, and then the levels of secreted TNFα were determined by ELISA. (E) Control U937 macrophages expressing a non-targeting CRISPR guide plasmid (black bars) and CRISPR-generated U937 cells KO of CD14 (purple and magenta bars) were infected with WT *L*. *pneumophila* 130b at a MOI of 20, and levels of secreted IL-6 (left) and TNFα (right) were determined 9 h later by ELISA. Graphs in (B) to (E) show the average cytokine levels (*n* = 3) pooled from three independent experiments, done in technical triplicate with standard errors. Asterisks indicate points at which the values for samples from KO cells were different from those for samples from CTL cells (**P < 0.01, ***P < 0.001, ****P < 0.0001, by Student’s *t* test).
(TIF)

S1 Table Reagents for shRNA and siRNA gene KD. (TIF)

S2 Table CRISPR/Cas9 gene KO design. (TIF)

S3 Table qRT-PCR primers for confirmation of shRNA KD. (TIF)

S4 Table CRISPR confirmation primers. (TIF)
