# Supplementary figures and images for: Human macrophages utilize a wide range of pathogen recognition receptors to recognize Legionella pneumophila, including Toll-Like Receptor 4 engaging Legionella lipopolysaccharide and the Toll-like Receptor 3 nucleic-acid sensor
Source: PLoS Pathog. 2021 Jul 19;17(7):e1009781. doi: 10.1371/journal.ppat.1009781 (PMC8321404; doi:10.1371/journal.ppat.1009781)

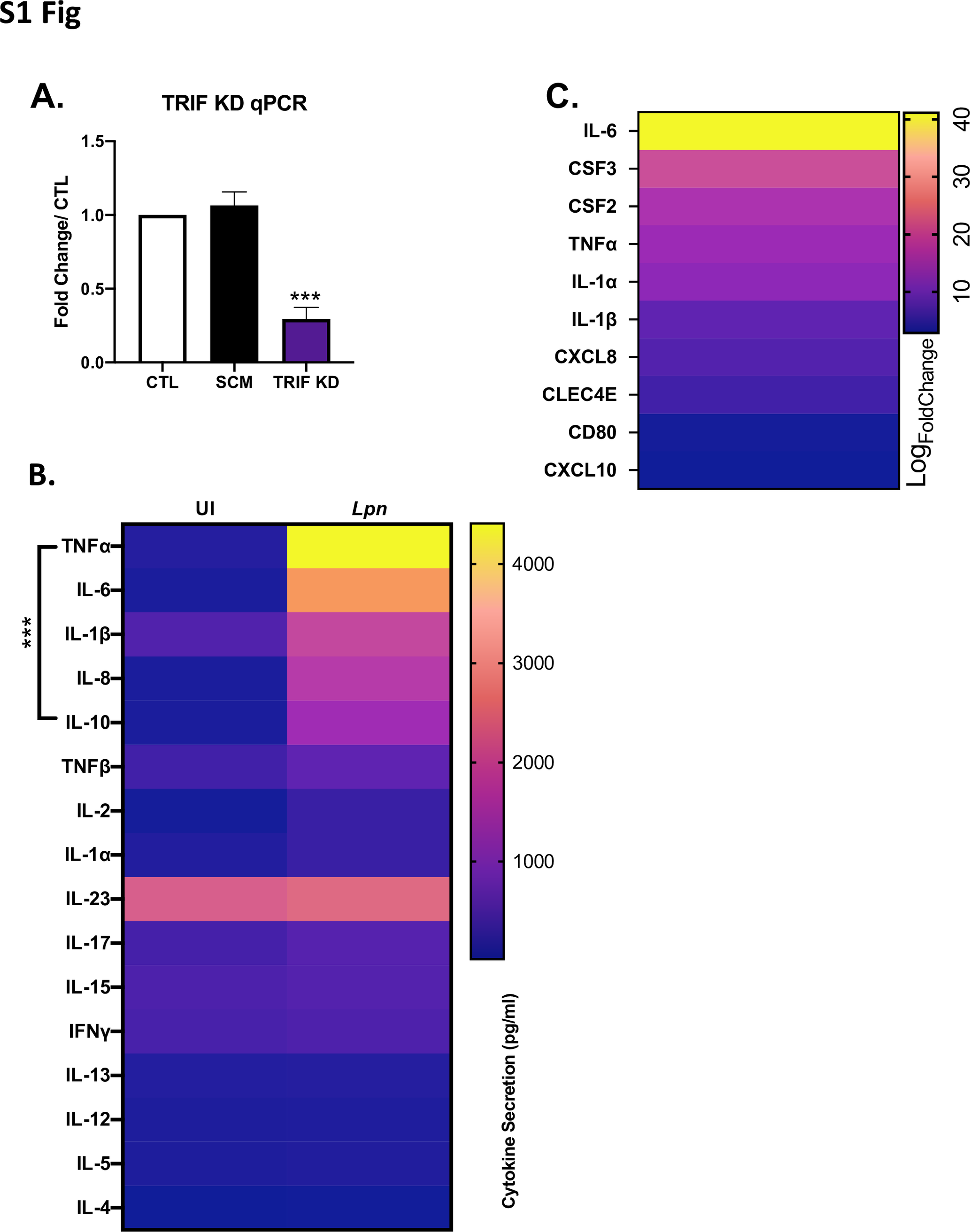

Supplement: S1 Fig — (A) qRT-PCR of the TRIF gene in non-transfected (CTL), scramble-transfected (SCM), and TRIF KD U937 cells was used to test KD efficiency. The value for the CTL was set to 1, and the values for the others is represented as fold-change compared to CTL. (B) U937 cells were either not infected (UI) or infected with strain 130b at a MOI of 20, and the levels of sixteen different cytokines in culture supernatants at 9 h post infection were then ascertained by multiplex ELISA analysis, with the pg/ml levels presented using the indicated color scheme. Multiplex represents the analysis of three pooled biological replicates (n = 3) each done in technical triplicate. (C) U937 cells were either not infected or infected with strain 130b at a MOI of 20, and the levels of cytokine and chemokine transcripts at 1 h post-infection were then determined using a Qiagen RT-PCR Array. Fold-changes in transcripts were calculated relative to uninfected U937 cells and are presented in log10 units using the indicated color scheme. All ten factors indicated here had a significant increase in expression upon infection (P < 0.05, by Student’s t test). Asterisks indicate five cytokines whose levels were significantly greater in the infected monolayers (***P < 0.001, by Student’s t test). (TIF) [file ppat.1009781.s001.tif]

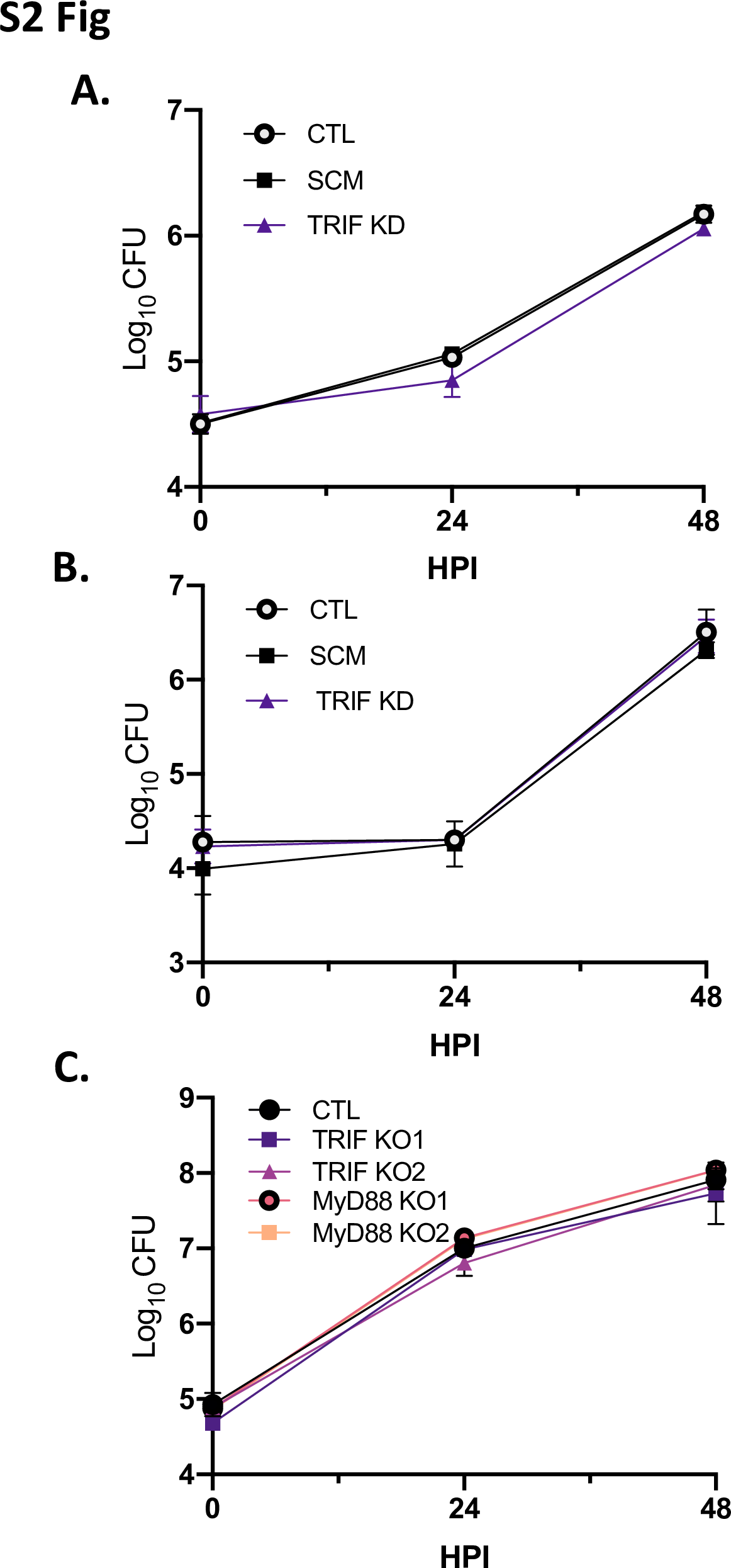

Supplement: S2 Fig — (A–B) Non-transfected U937 cells (CTL) or U937 cells containing either an shRNA targeting TRIF (TRIF KD) or a non-targeting scramble shRNA (SCM) were infected with strain 130b (A) or Philadelphia-1 (B) at a MOI of 0.5, and then bacterial CFU were determined by plating at 0, 24, and 48 h post-infection (HPI). (C) U937 cell macrophages containing either a mutation in the TRIF gene (TRIF KO1, TRIF KO2) or MyD88 gene (MyD88 KO1, MyD88 KO2) or a non-targeting CRISPR Guide plasmid (CTL) were infected with strain 130b at a MOI of 20 and CFU determined as above. Data represent the mean CFU (on log10 scale) and standard error for triplicate samples and for each panel are representative of three independent experiments. (TIF) [file ppat.1009781.s002.tif]

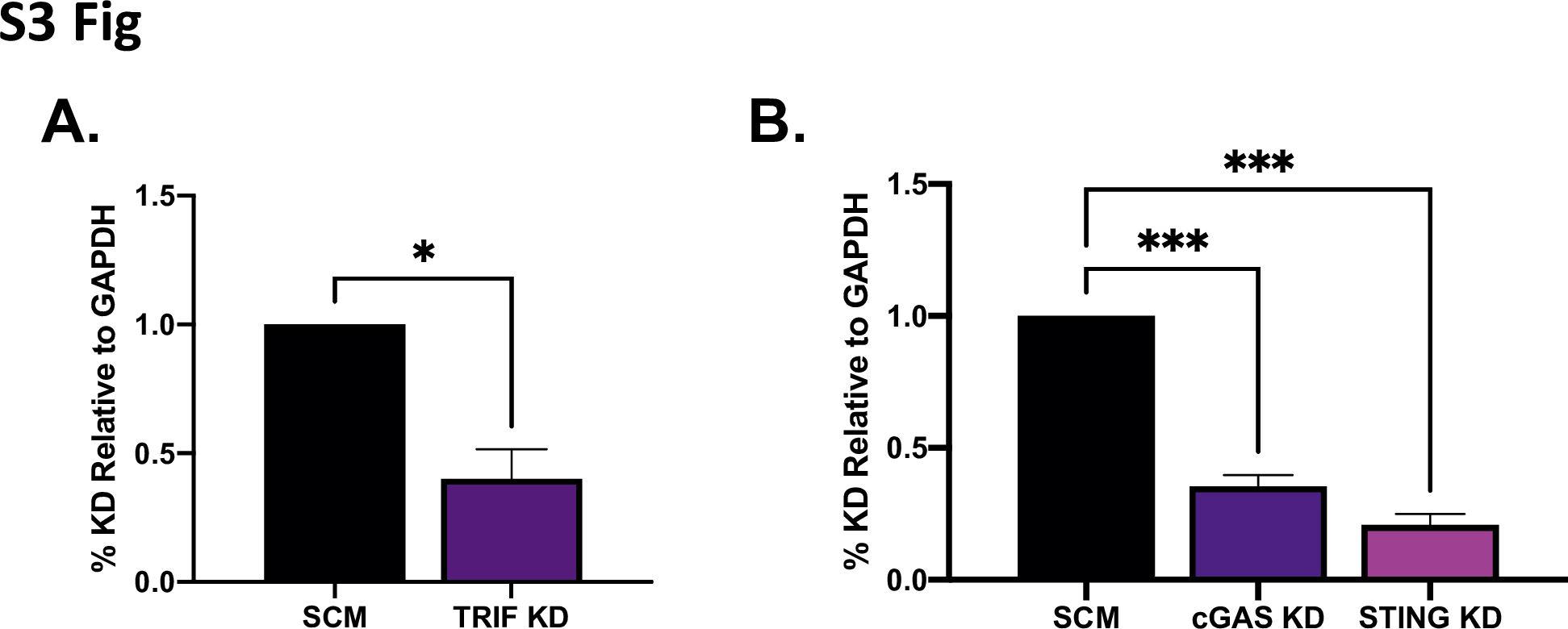

Supplement: S3 Fig — (A) Human PBMC-derived macrophages containing either an siRNA targeting TRIF (TRIF KD) or a non-targeting scramble siRNA (SCM) were examined by immunoblot for the presence of TRIF protein or GAPDH. The KD was done in technical duplicate and then assayed on two occasions. The mean fluorescent intensity quantification of the western blot was then used to determine the specific loss of TRIF. (B) U937 macrophages containing either an shRNA targeting cGAS (cGAS KD), STING (STING KD) or a non-targeting scramble shRNA (SCM) were examined by immunoblot for the presence of cGAS/STING protein or GAPDH. The KD was done in technical triplicate and then assayed on two occasions. The mean fluorescent intensity quantification of the western blot was then used to determine the specific loss of cGAS/STING was used to test KD efficiency. In (A-B), the value for the SCM was set to 1, and values for the others is represented as fold-change compared to CTL. Asterisks indicate points at which the values for samples from KD cells were significantly different from those for samples from SCM cells (* P < 0.05, ***P < 0.001, by Student’s t test). (TIF) [file ppat.1009781.s003.tif]

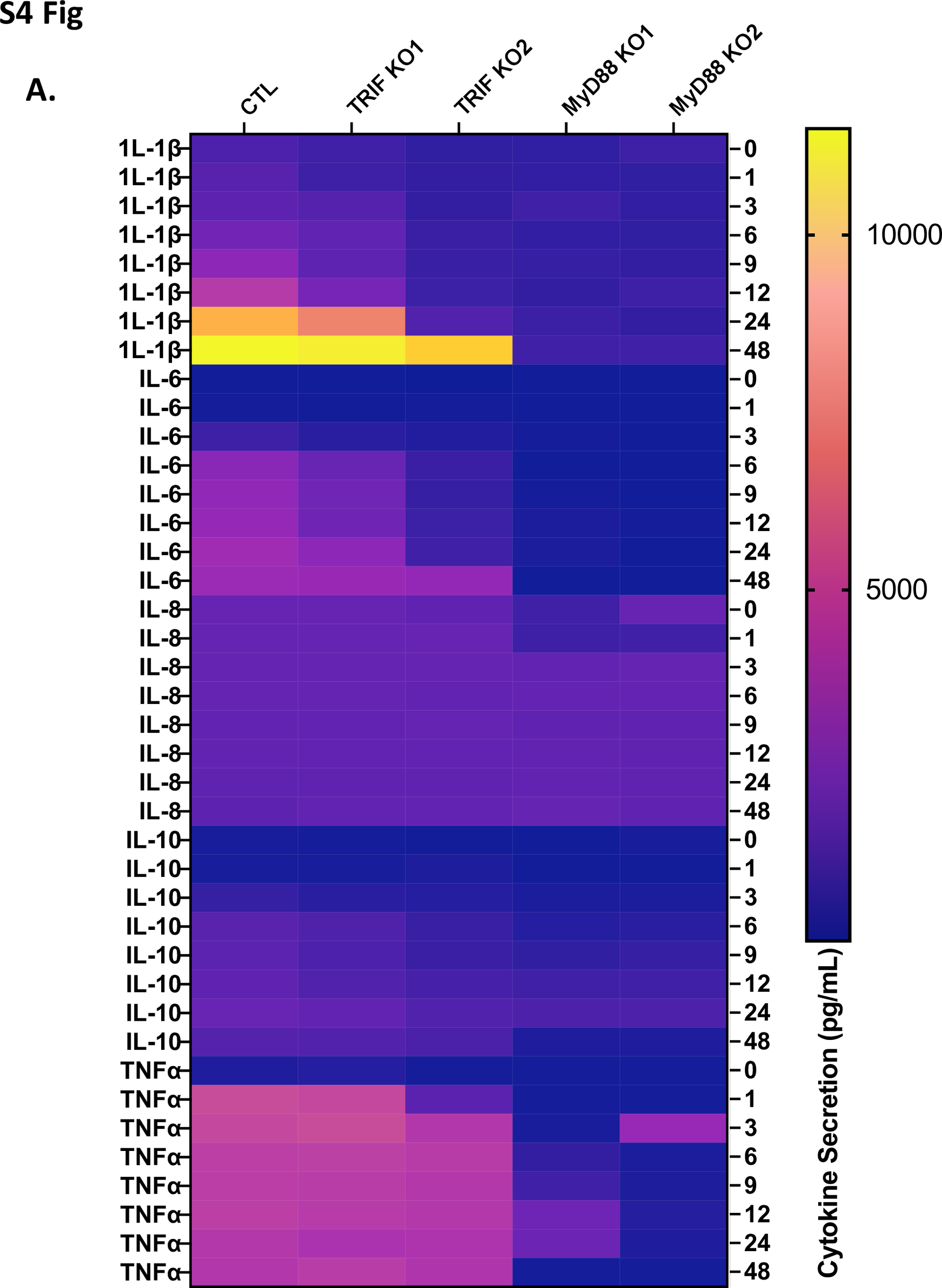

Supplement: S4 Fig — Control, TRIF KO, and MyD88 KO U937 cells were infected with L. pneumophila strain 130b at a MOI of 20, and the secreted levels of IL-1β, IL-6, IL-8, IL-10 and TNFα (as indicated on left side) at 0, 1, 3, 6, 9, 12, 24, and 48 h post infection (right side) were ascertained by multiplex cytokine ELISA, with pg/ml levels presented using the indicated color scheme (far right side). The multiplex system represents analysis of three pooled biological replicates (n = 3) each done in technical triplicate. (TIF) [file ppat.1009781.s004.tif]

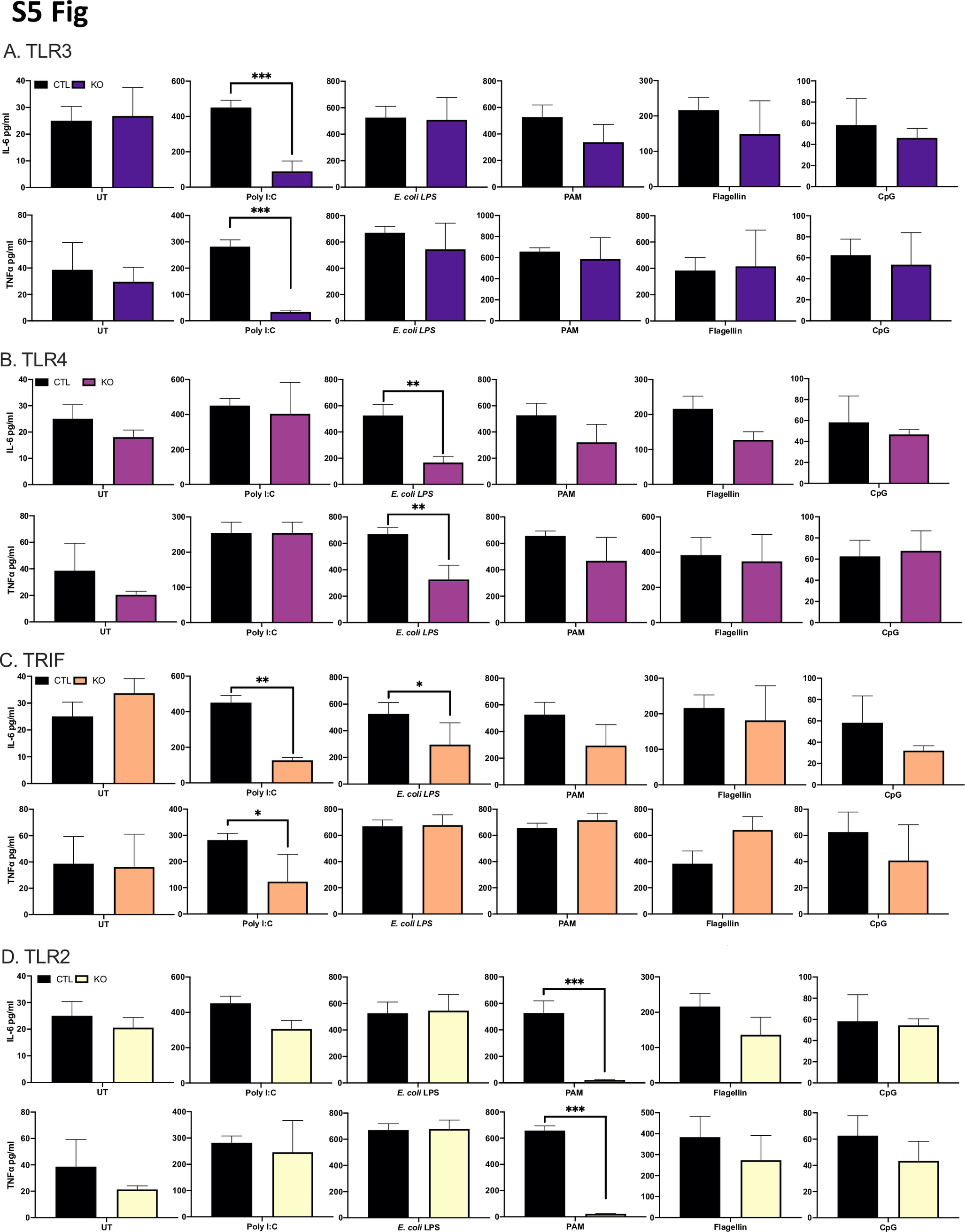

Supplement: S5 Fig — U937 cells expressing a non-targeting CRISPR guide plasmid (CTL, black bars) and U937 cells containing a CRISPR/Cas9-generated mutation (KO1) in either TLR3 (A), TLR4 (B), TRIF (C) and TLR2 (D) were either untreated (UT) or treated with Poly I:C, E. coli LPS, PAM, flagellin, or CpG, and the levels of secreted IL-6 and TNFα at 12 h post treatment were then determined by ELISA. Secreted cytokine levels (pg/ml) were calculated relative to serial dilution of the recombinant cytokine standard. Graphs show the average cytokine levels pooled from two independent experiments, done in technical triplicate, with standard errors. For ease of comparing the effects of the different treatments on a given KD/KO, the treated CTL result was presented in each row. Asterisks indicate points at which the values for samples from the KO/KD cells were significantly different from those for samples from CTL cells (*P < 0.05, **P < 0.01, ***P < 0.001, by Student’s t test). (TIF) [file ppat.1009781.s005.tif]

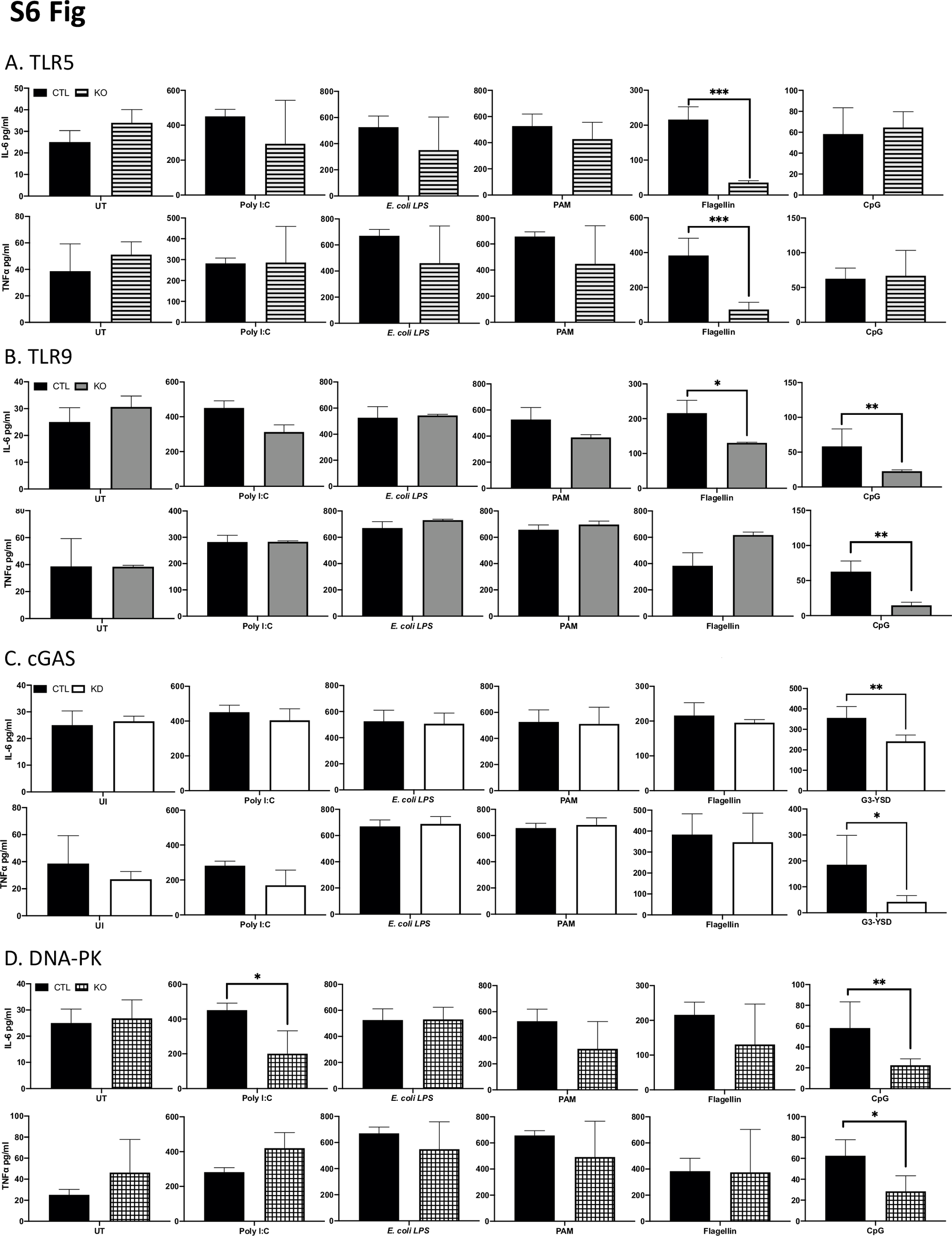

Supplement: S6 Fig — U937 cells expressing a non-targeting CRISPR guide plasmid (CTL, black bars) and U937 cells containing a CRISPR/Cas9-generated mutation (KO1) in either TLR5 (A), TLR9 (B), cGAS (C), or DNA-PK (D) were either untreated (UT) or treated with Poly I:C, E. coli LPS, PAM, flagellin, CpG, or G3-YSD, and the levels of secreted IL-6 and TNFα at 12 h post treatment were then determined by ELISA. Secreted cytokine levels (pg/ml) were calculated relative to serial dilution of the recombinant cytokine standard. Graphs show the average cytokine levels pooled from two independent experiments, done in technical triplicate, with standard errors. For ease of comparing the effects of the different treatments on a given KD/KO, the treated CTL result was presented in each row. Asterisks indicate points at which the values for samples from the KO/KD cells were significantly different from those for samples from CTL cells (*P < 0.05, **P < 0.01, ***P < 0.001, by Student’s t test). (TIF) [file ppat.1009781.s006.tif]

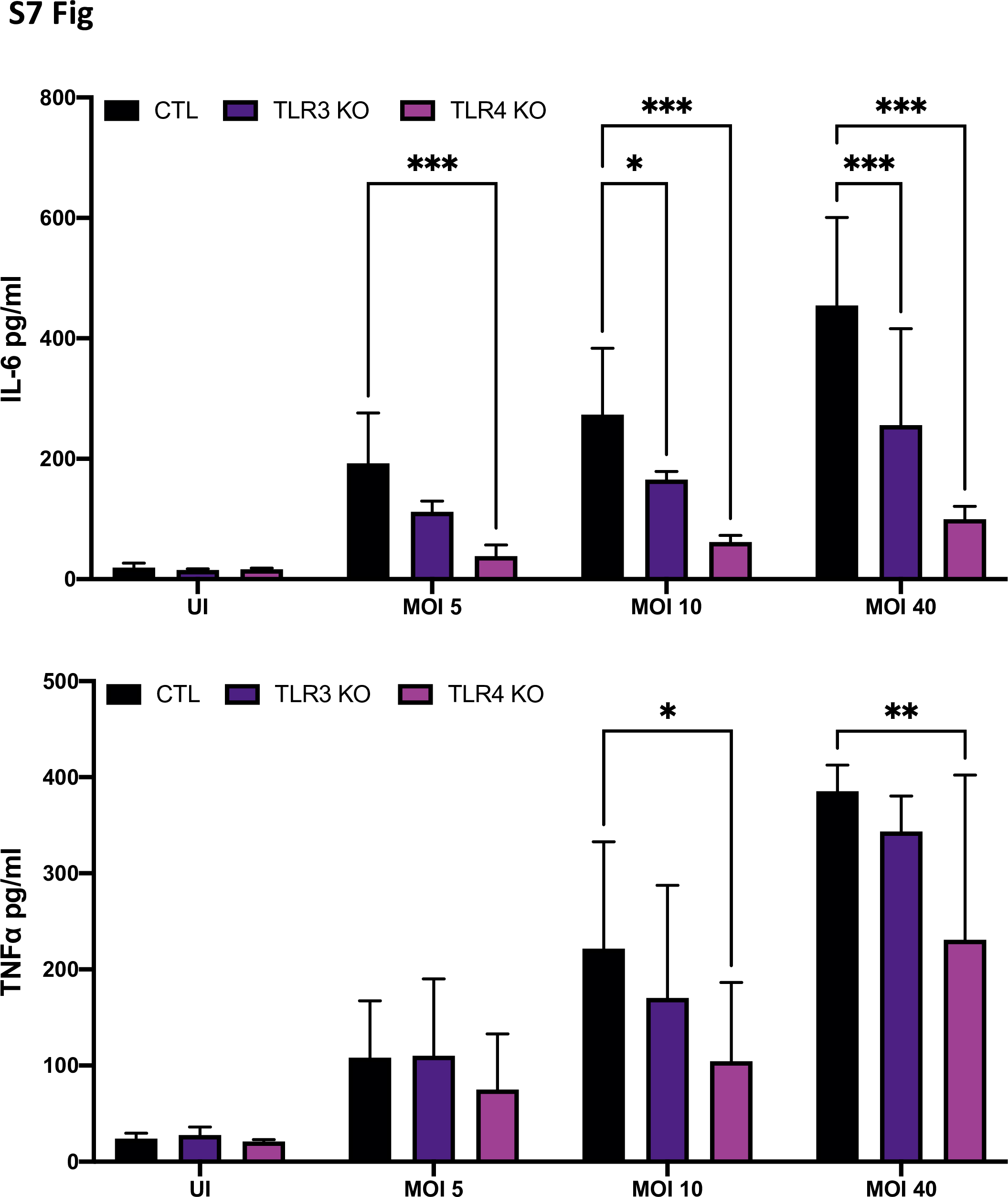

Supplement: S7 Fig — U937 cells expressing a non-targeting CRISPR guide plasmid (CTL, black bars) and U937 cells with a CRISPR-generated KO of TLR3 or TLR4 (KO, purple and magenta bars) were either not infected (UI) or infected with L. pneumophila at a MOI of 5, 10, or 40 as indicated, and the levels of secreted IL-6 and TNFα at 9 h post-infection were then determined by ELISA. The cytokine levels (pg/ml) were calculated relative to serial dilution of recombinant cytokine controls. Graphs show the average cytokine levels (n = 2) pooled from two independent experiments, done in technical triplicate, with standard errors. Asterisks indicate points at which the values for samples from KO cells were significantly different from those of CTL cells (*P < 0.05, **P < 0.01, ***P < 0.001, by Student’s t test). (TIF) [file ppat.1009781.s007.tif]

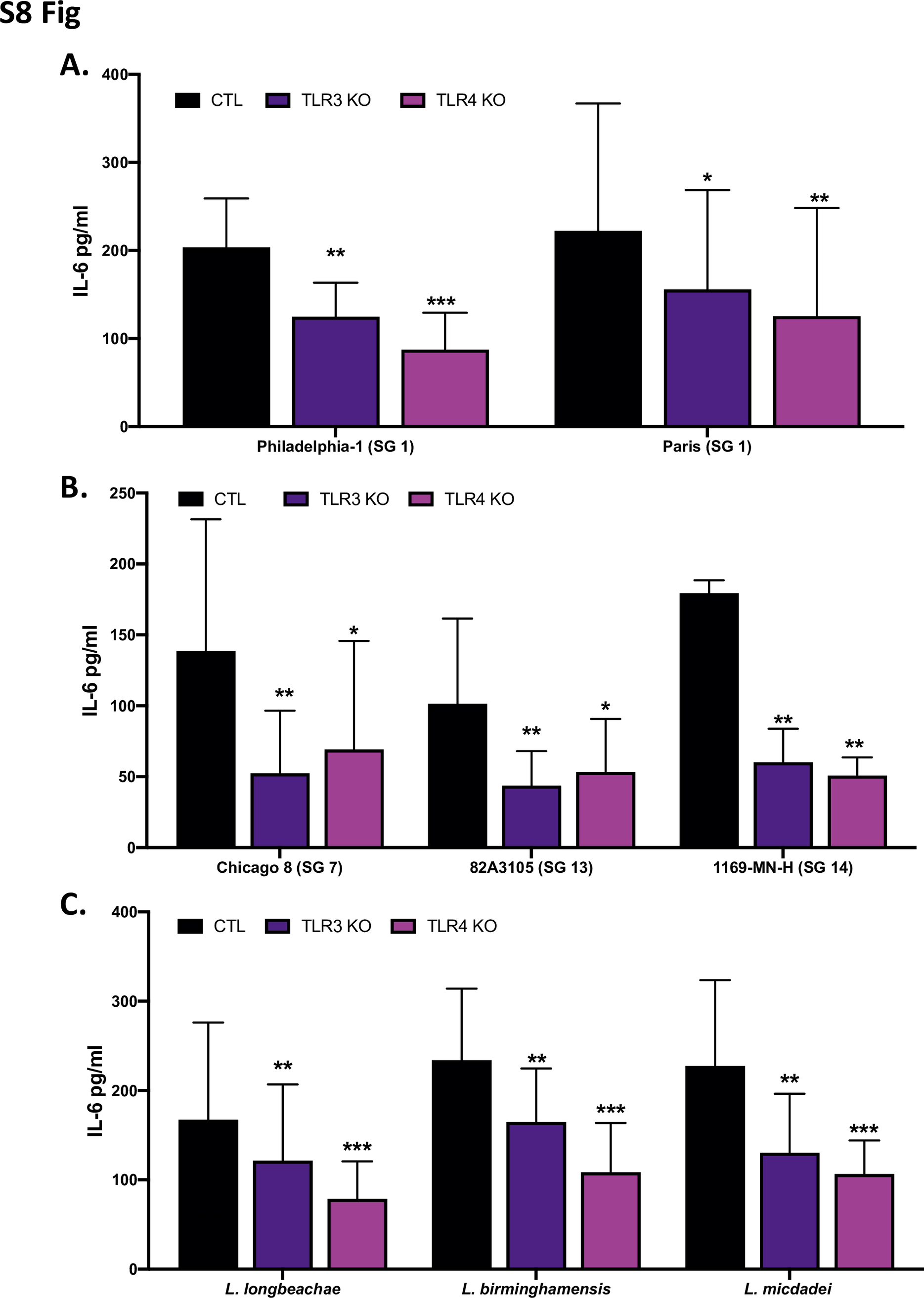

Supplement: S8 Fig — U937 cells expressing a non-targeting CRISPR guide plasmid (CTL, black bars) and U937 cells with a CRISPR-generated KO of TLR3 or TLR4 (KO, purple and magenta bars) were infected with L. pneumophila serogroup (SG) 1 strains Philadelphia-1 and Paris (A), L. pneumophila strains representing SG 7, 13, and 14 (B), and strains representing the Legionella species L. longbeachae, L. birminghamensis, and L. micdadei (C) at a MOI of 20, and the levels of secreted IL-6 at 9 h post-infection were then determined by ELISA. The cytokine levels (pg/ml) were calculated relative to serial dilution of recombinant cytokine controls. Graphs show the average cytokine levels (n = 3) pooled from three independent experiments, done in technical triplicate, with standard errors. Asterisks indicate points at which the values for samples from KO cells were significantly different from those of CTL cells (*P < 0.05, **P < 0.01, ***P < 0.001, by Student’s t test). (TIF) [file ppat.1009781.s008.tif]

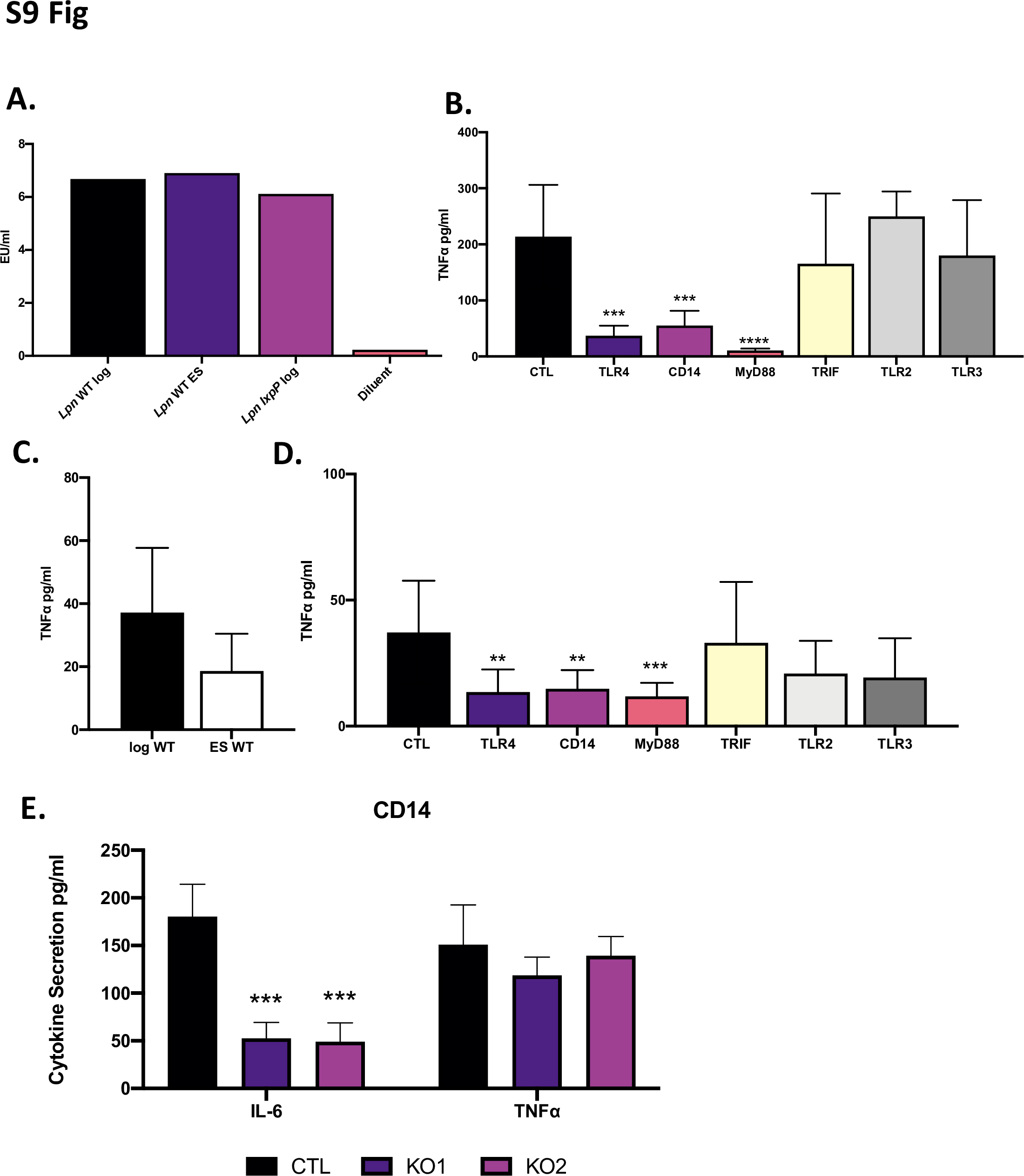

Supplement: S9 Fig — (A) LPS purified from 5-ml samples of log-phage (log) or early-stationary phase (ES) cultures of either wildtype L. pneumophila strain 130b (WT) or a lpxP mutant derivative of strain 130b (lpxP) were examined using the LAL-assay. The diluent used to resuspend the LPS was used as a negative control and also tested. The concentrations of LPS in each of the L. pneumophila samples (in technical duplicate) were then determined by comparison to a standard curve generated using the results obtained with the E. coli LPS. LPS concentrations are expressed as endotoxin units (EU) per ml, where one EU equals 0.1 to 0.3 ng LPS/ml, as per the manufacturer’s protocol. As depicted here, the LPS in the L. pneumophila samples corresponded to approx. 1–2 ng of E. coli LPS. Based on these data, all subsequent experiments utilized a defined amount of LPS. (B) CTL U937 cells were treated for 12 h with ~100 ng/μl of L. pneumophila LPS purified from either log phase (log) or early-stationary phase (ES) cultures of wildtype strain 130b (WT), and then secreted TNFα levels determined by ELISA. (C—D) U937 macrophages expressing a non-targeting CRISPR guide plasmid (CTL) and CRISPR-generated U937 KOs lacking either TLR4, CD14, MyD88, TRIF, TLR2 and TLR3 were treated for 12 h with either E. coli LPS at 1 ng/μl (C), or log-phase L. pneumophila LPS at 100 ng/μl, and then the levels of secreted TNFα were determined by ELISA. (E) Control U937 macrophages expressing a non-targeting CRISPR guide plasmid (black bars) and CRISPR-generated U937 cells KO of CD14 (purple and magenta bars) were infected with WT L. pneumophila 130b at a MOI of 20, and levels of secreted IL-6 (left) and TNFα (right) were determined 9 h later by ELISA. Graphs in (B) to (E) show the average cytokine levels (n = 3) pooled from three independent experiments, done in technical triplicate with standard errors. Asterisks indicate points at which the values for samples from KO cells were different from those for samples from CTL cell [file ppat.1009781.s009.tif]

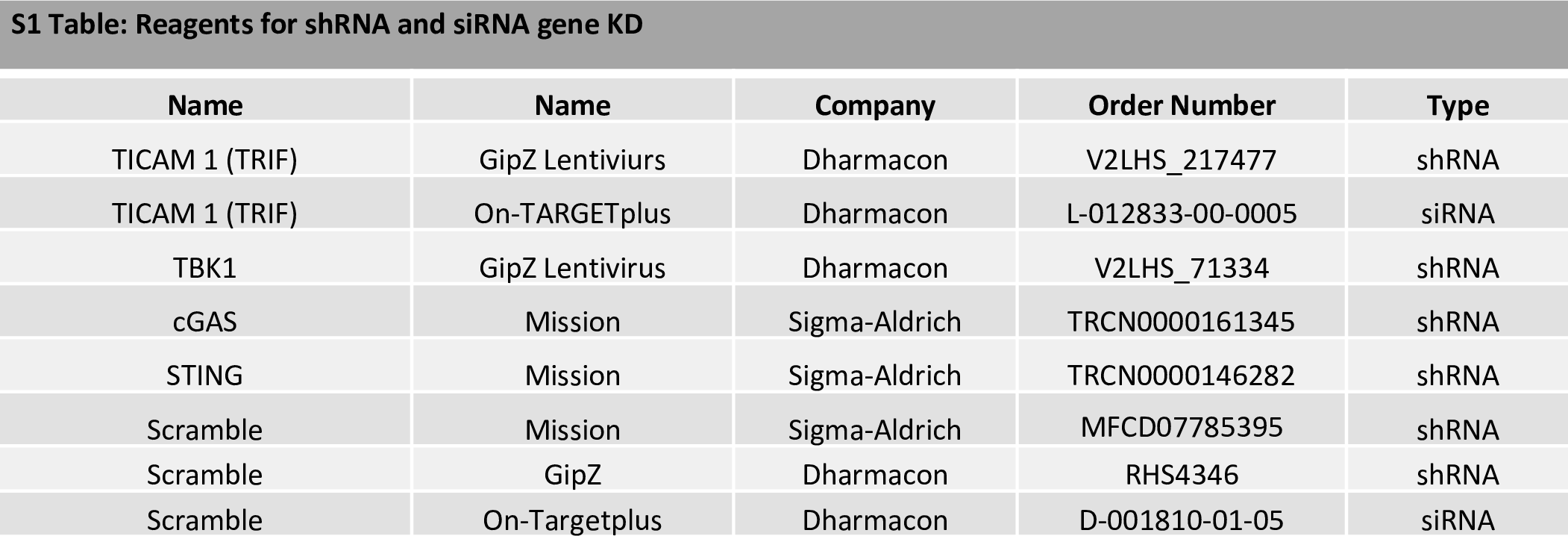

Supplement: S1 Table — (TIF) [file ppat.1009781.s010.tif]

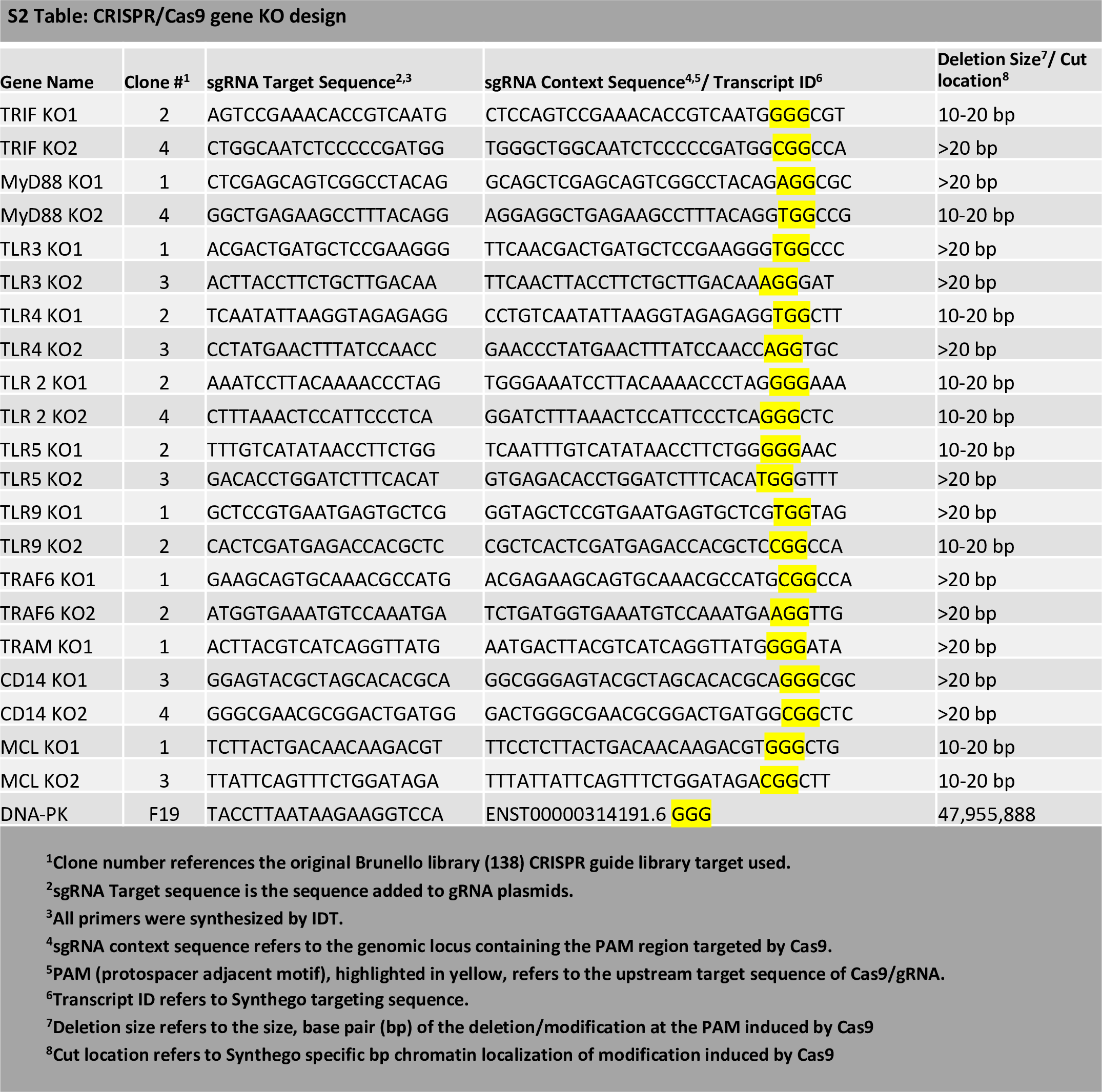

Supplement: S2 Table — (TIF) [file ppat.1009781.s011.tif]

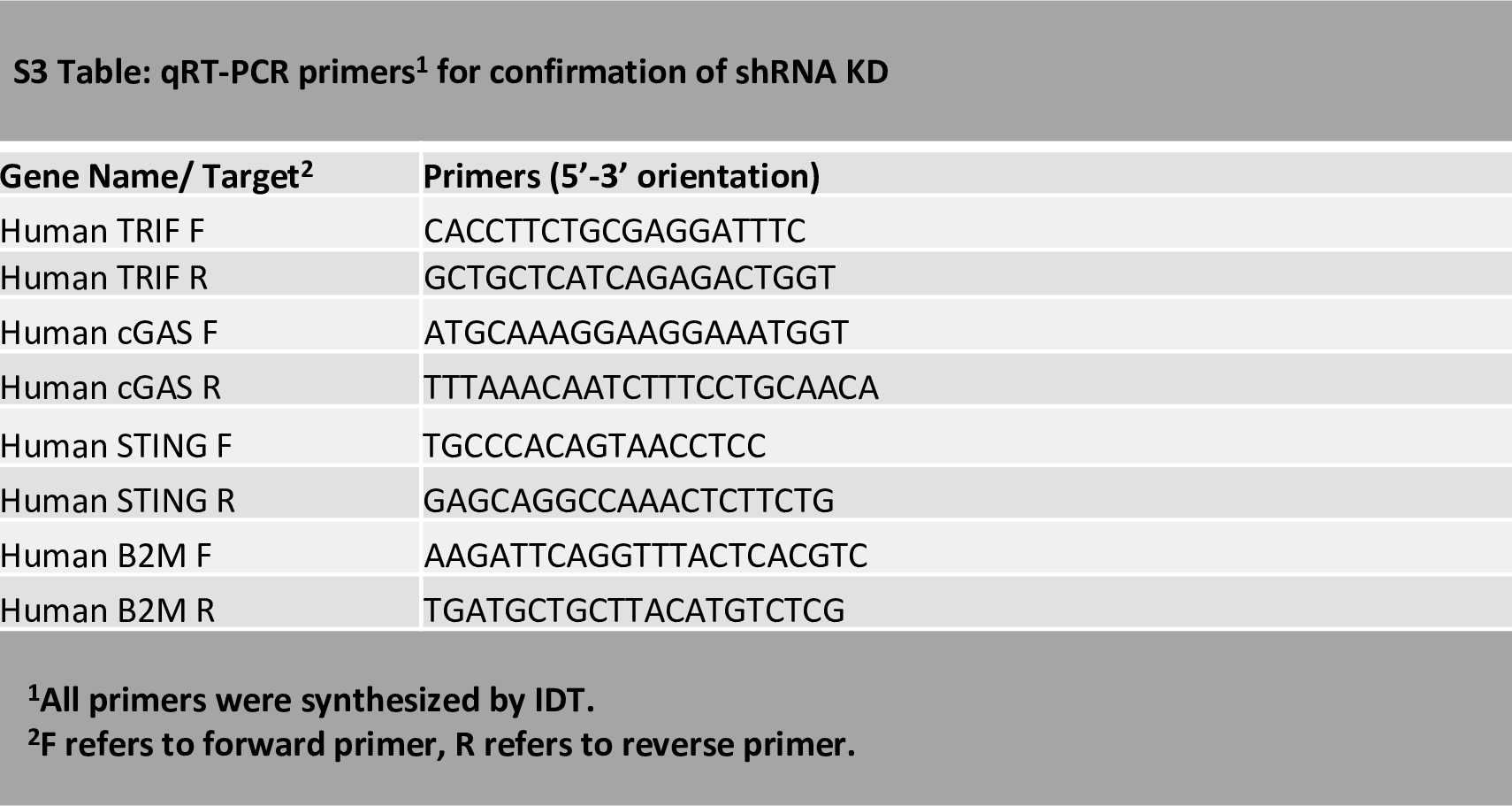

Supplement: S3 Table — (TIF) [file ppat.1009781.s012.tif]

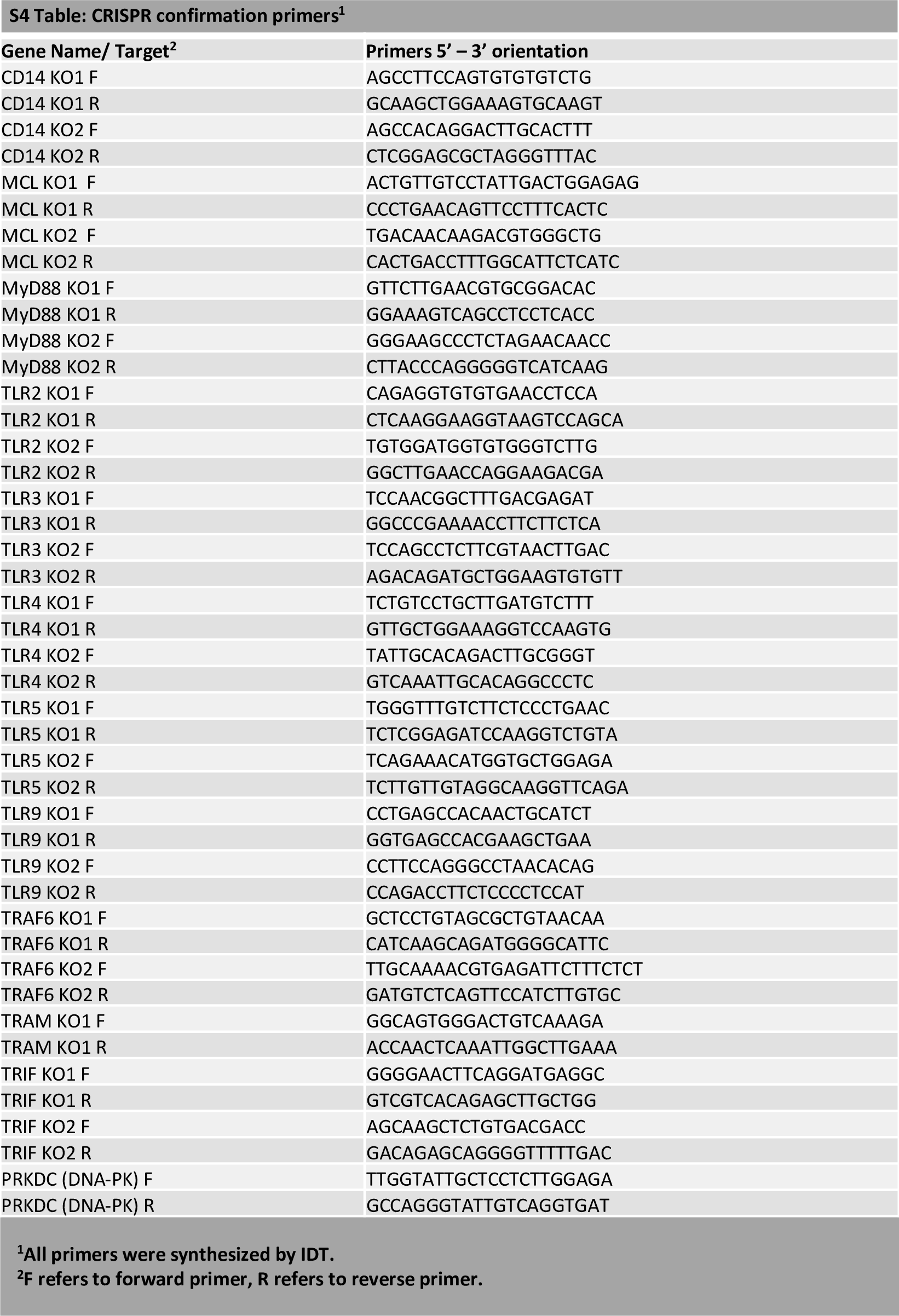

Supplement: S4 Table — (TIF) [file ppat.1009781.s013.tif]
